# Supplementary material for: Proximity proteomics identifies PAK4 as a component of Afadin–Nectin junctions
Source: Nat Commun. 2021 Sep 7;12:5315. doi: 10.1038/s41467-021-25011-w (PMC8423818; doi:10.1038/s41467-021-25011-w)
Supplement: Supplementary file 1 — Supplementary Information [file 41467_2021_25011_MOESM1_ESM.pdf]

SUPPLEMENTARY DATA LIST

Supplementary Figure S1. Afadin knockdown does not alter the localization of DLG5 and Scribble.

Supplementary Figure S2. PAK4 inhibition with PF-3758309 enhanced the development of cell-cell junctions with higher PAK4/afadin content compared to p120 catenin.

Supplementary Figure S3. PAK4 knockdown does not alter localization of β-catenin and PAK4/Afadin colocalization with ZO1.

Supplementary Figure S4. Afadin dependent localization of PAK4 to cell-cell junctions.

Supplementary Figure S5. ROCK inhibition does not alter localization of beta-catenin.

Supplementary Figure S6. N-terminal basic region is required for PAK4 localization at cell-cell junctions. Contractibility dependent localization of Afadin at cell-cell junctions.

Supplementary Dataset T1. BioID list of Afadin proximal proteins in HEK293 cells.

Supplementary Dataset T2. BioID list of PAK4, Cdc42 proximal proteins and PAK4 affinity in U2OS cells.

Supplementary Dataset T3. Putative PAK4 substrates by phospho-proteomic analysis.

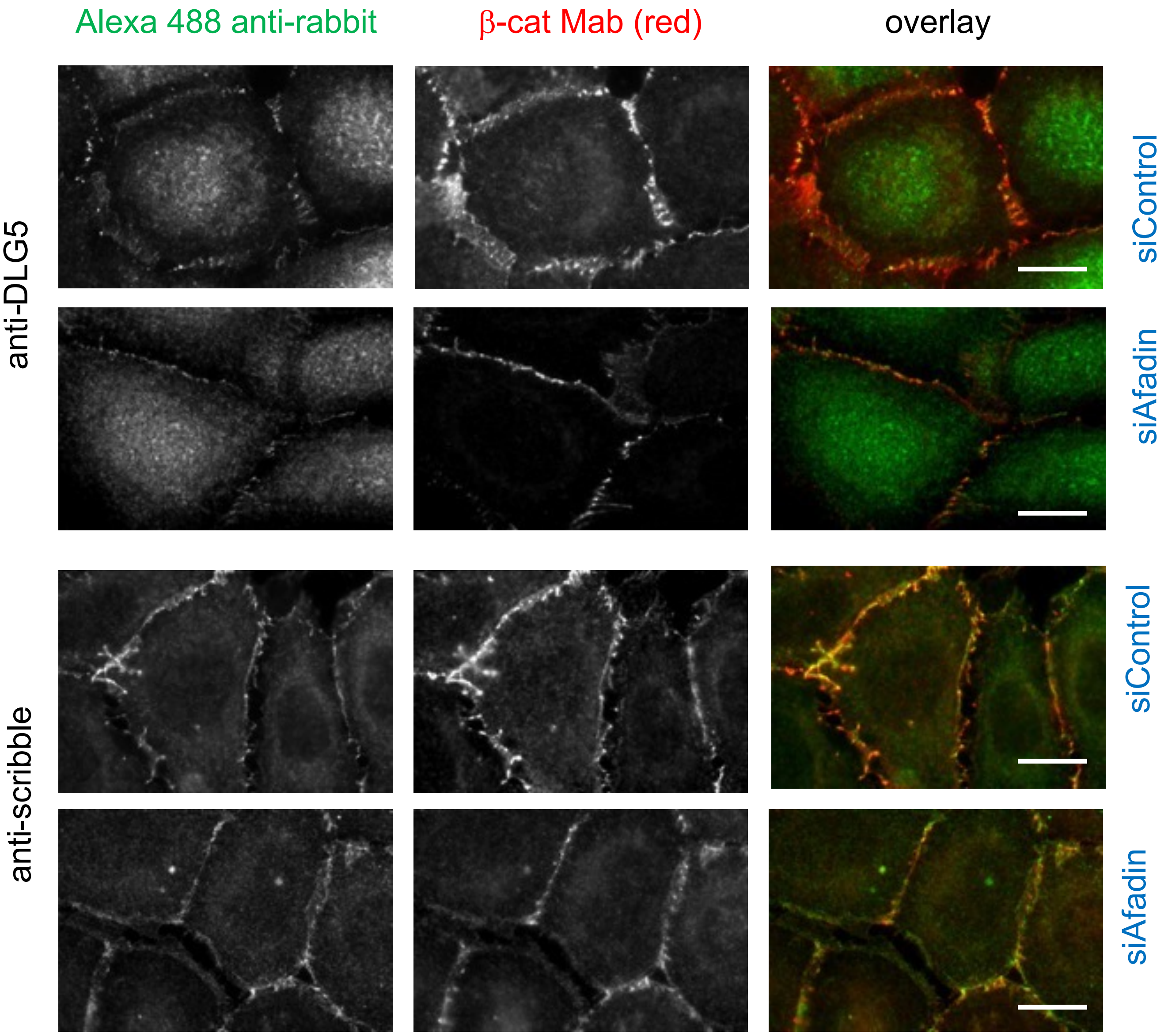

Supplementary Figure S1. Afadin knockdown does not alter the localization of DLG5 and Scribble. Afadin knockdown does not alter the localization of DLG5 and Scribble. U2OS cells were treated with Afadin and control scrambled siRNA and fixed 48 hours later. Cells were stained using rabbit antibodies specific for DLG5 and Scribble and mouse antibodies for b-catenin, as a junction marker.

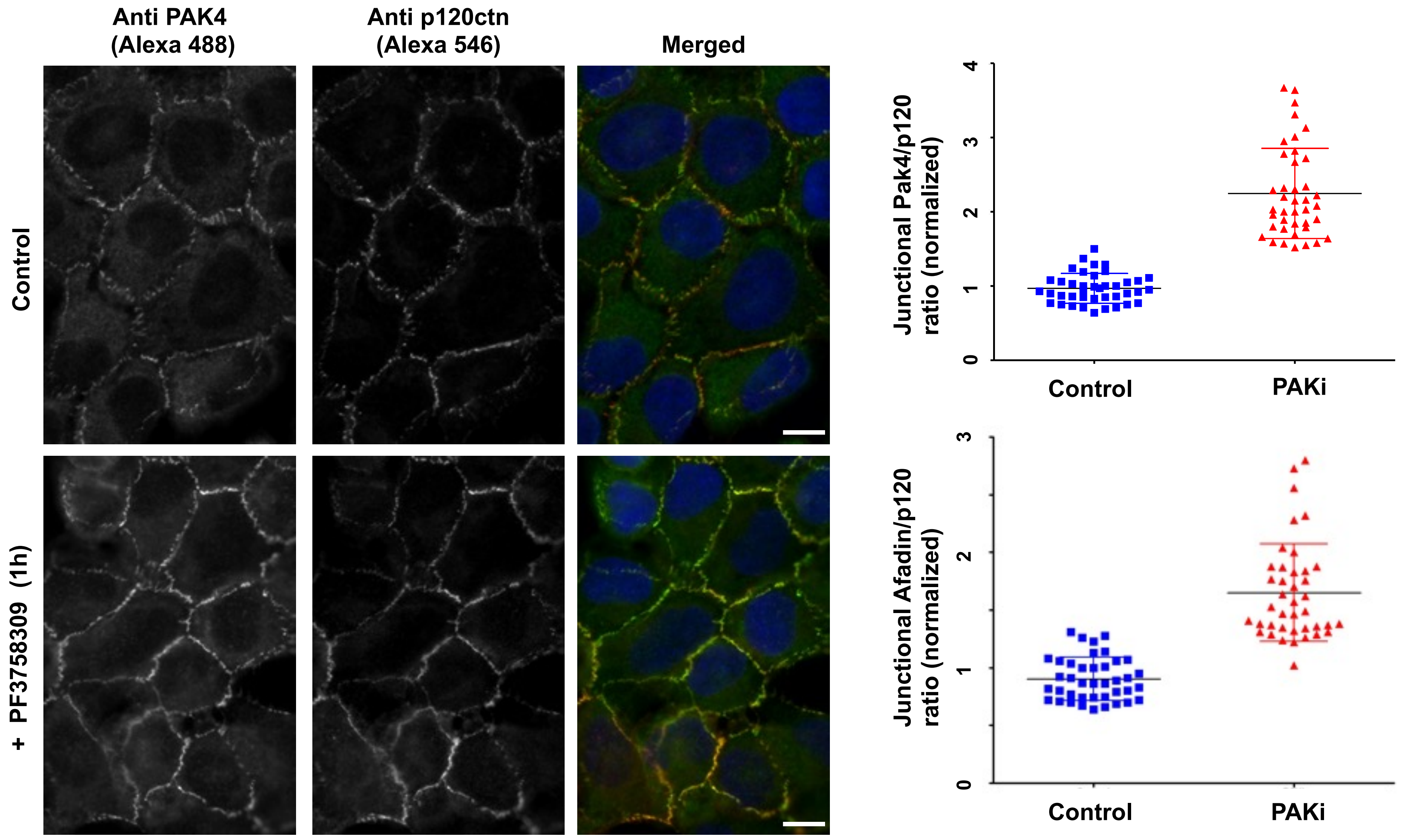

Supplementary Figure S2. PAK4 inhibition with PF-3758309 enhanced the development of cell-cell junctions with higher PAK4/afadin content compared to p120 catenin. U2OS cells were grown to 80% confluence on uncoated glass coverslips. Then rinsed in warm calcium free PBS and incubated in serum free DME +5 mM EGTA for 45 mins, Media containing 5% serum (1.8 mM calcium) was added for 45 min to allow reattachment, before addition of inhibitor/DMSO for 2 hrs. Cells fixed in methanol (100%) and stained for anti-PAK4 or anti-Afadin (Alexa 488) and mouse anti-p120ctn Mab (Alexa 546). The ratio of PAK4 or Afadin intensity to p120ctn, normalised to the background/per standard unit length, was measured (n=30) over 3 separate experiments. The SD of the mean is indicated. 10 µm scale bars. Source data are provided as a Source Data file.

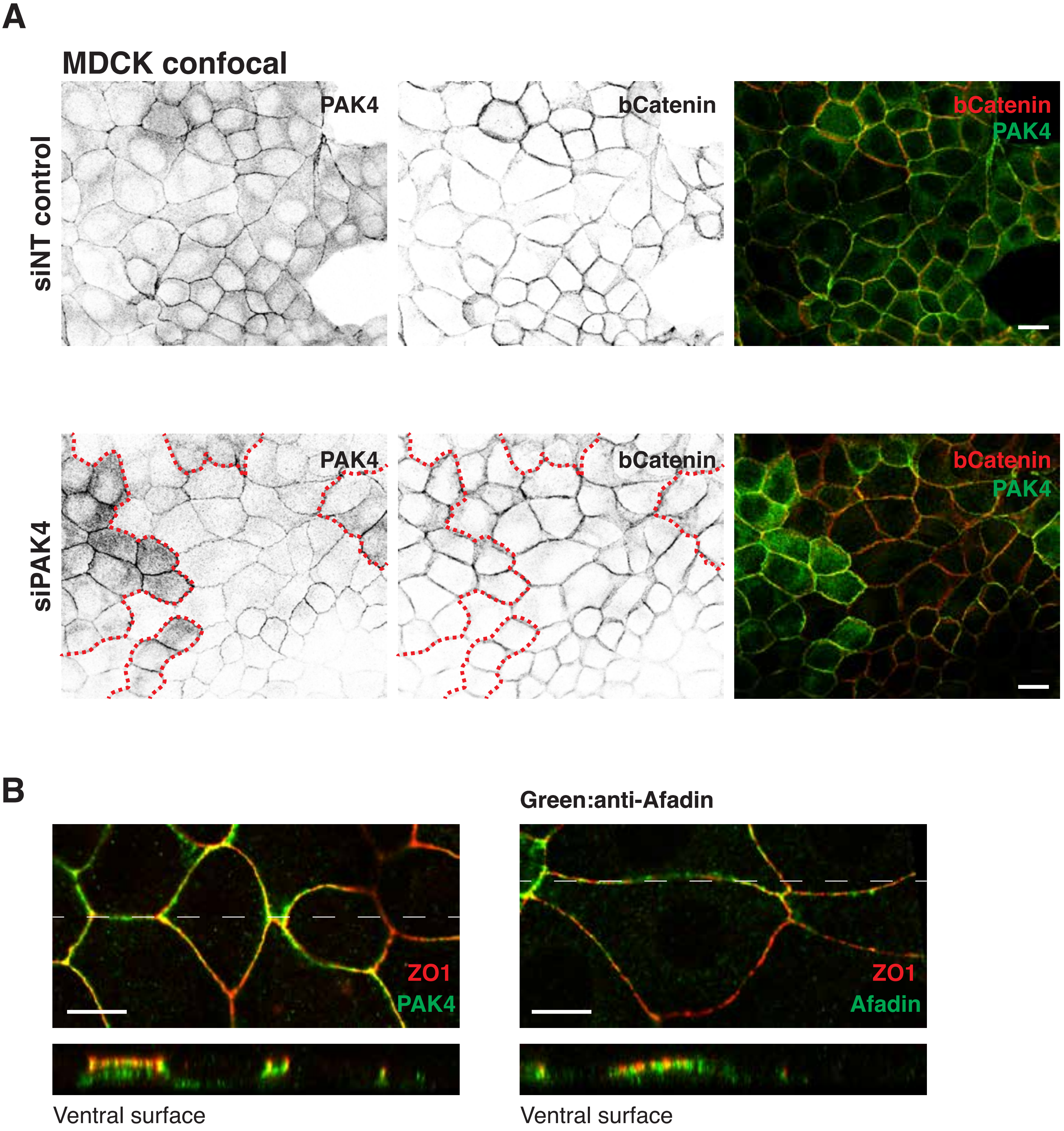

Supplementary Figure S3. PAK4 knockdown does not alter localization of β-catenin and PAK4/Afadin colocalization with ZO1 (A) MDCK cells were treated with non-targeting (NT) or PAK4 siRNA for 72h as indicated. Following fixation in methanol, the cells were immuno-stained for PAK4 (rabbit antibody)/beta-catenin (mouse antibody) as indicated. Confocal images were taken at 60X magnification. 20 μm scale bars. (B) Sub-confluent (2 day) cells were stained using rabbit antibodies specific for PAK4 and Afadin and rat antibody specific for ZO1 (60X objective). Z-axis projections shows the apico-basal disposition along the dotted line indicated above. 10 μm scale bars.

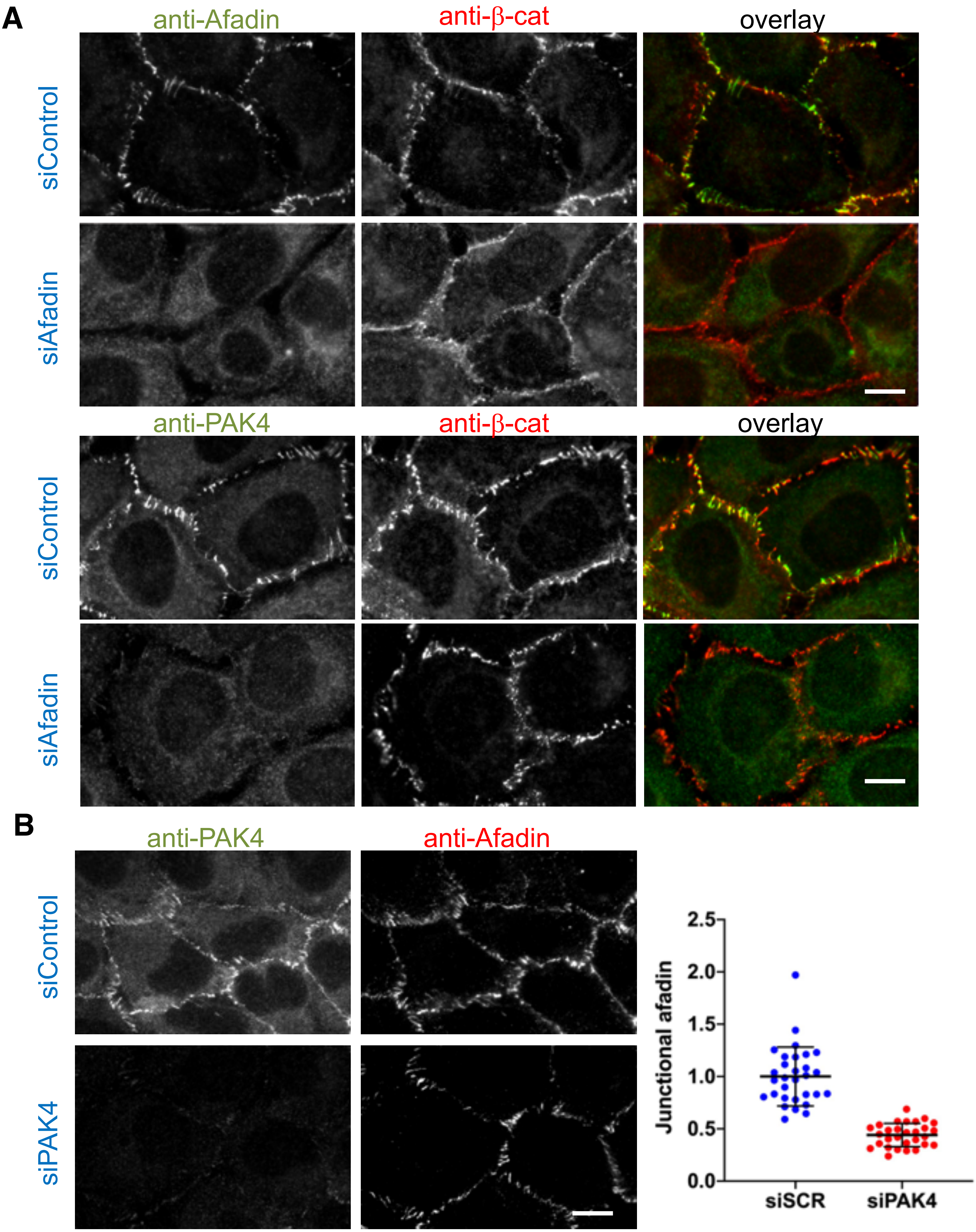

Supplementary Figure S4. Afadin dependent localization of PAK4 to cell-cell junctions. U2OS cells were treated with Afadin or PAK4 siRNA for 48 hrs as indicated (A) Following fixation in methanol, the cells were immuno-stained for PAK4, Afadin or b-catenin as indicated. (B) Scatter plot showing protein levels derived from immuno-fluorescent images. The junctional fluorescence signal was calculated for a standard area along the junction normalized for local background (non-junction) signal and recorded as a ratio to average (n=30). The data points from two independent experiments are displayed with bars indicating standard deviation from the mean. 10 μm scale bars. Source data are provided as a Source Data file.

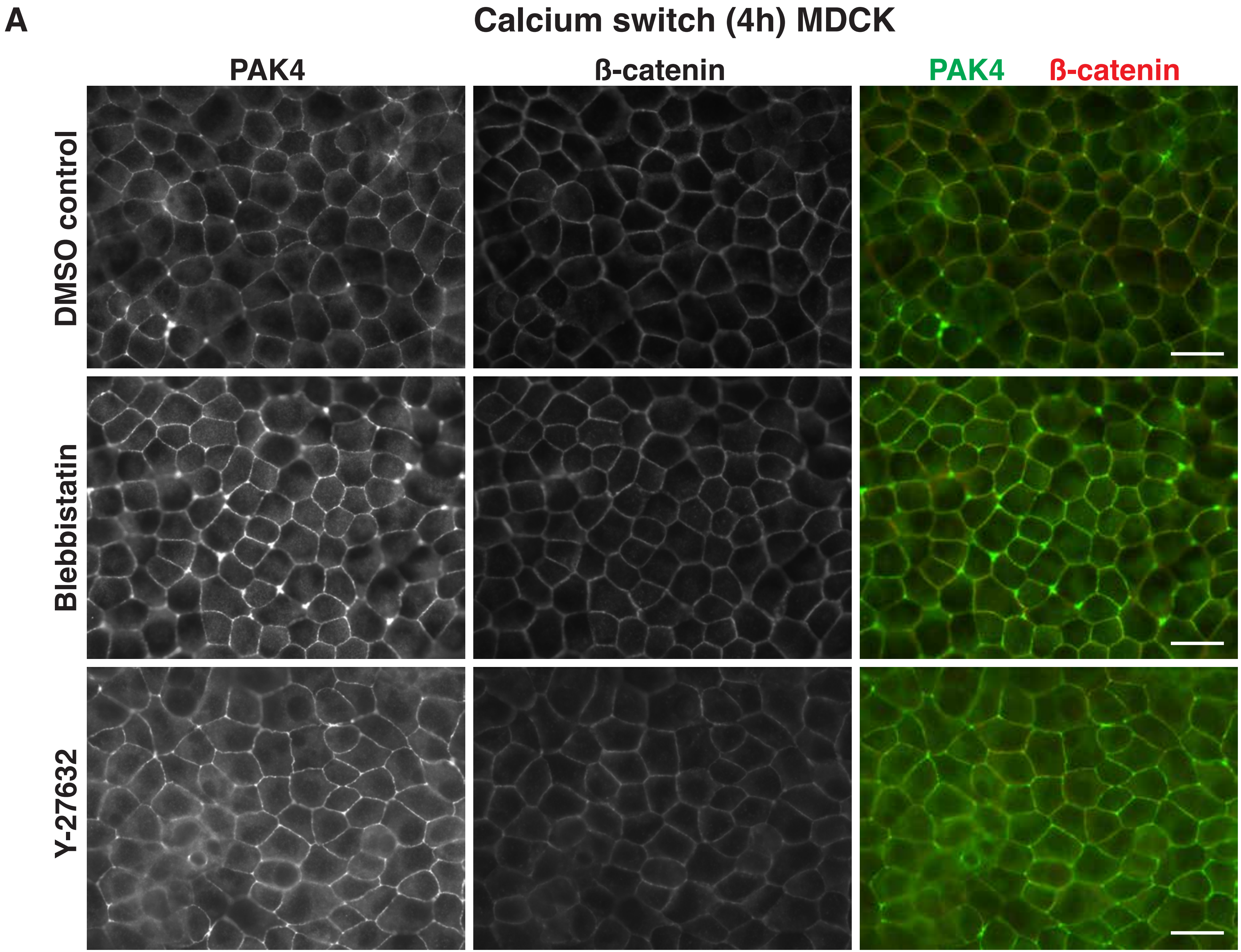

Supplementary Figure S5. ROCK inhibition does not alter localization of beta-catenin.
Calcium switch assay with confluent MDCK cells on coverslips, stained for PAK4 (rabbit antibody) or beta-catenin (mouse antibody) after 4 hrs recovery in the presence of control DMSO or the indicated inhibitors. Images were taken with a wide-field microscope at 630X magnification. 20 µm scale bars.

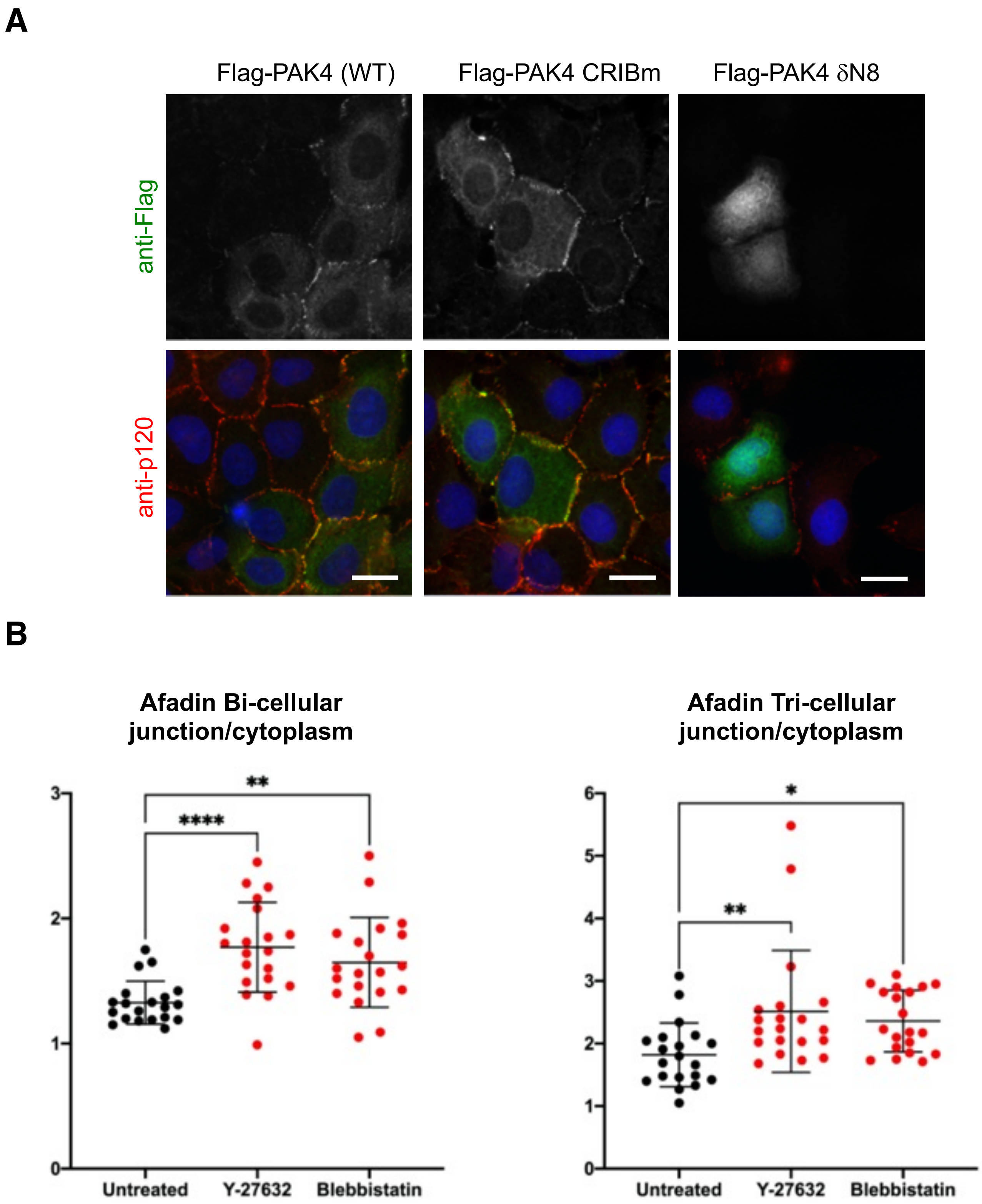

Supplementary Figure S6. N-terminal basic region is required for PAK4 localization at cell-cell junctions. Contractibility dependent localization of Afadin at cell-cell junctions.

(A) U2OS cells were transiently transfected with FLAG-PAK4 constructs as indicated. Cells fixed and stained with rabbit anti-FLAG (green) and anti-p120ctn Mab (red). 20 µm scale bars. (B) As in Figure 5C and D. Scatter plots of Afadin junctional protein levels (n=20). The data points from two independent experiments are displayed with bars indicating standard deviation from the mean and analyzed using an Ordinary one-way ANOVA test. Source data are provided as a Source Data file.
