# Supplementary material for: Proximity proteomics identifies PAK4 as a component of Afadin–Nectin junctions
Source: Nat Commun. 2021 Sep 7;12:5315. doi: 10.1038/s41467-021-25011-w (PMC8423818; doi:10.1038/s41467-021-25011-w)
Supplement: Supplementary file 3 — Description of Additional Supplementary Files [file 41467_2021_25011_MOESM3_ESM.pdf]

Description of Additional Supplementary Files

File Name: Supplementary Data 1
Description: BioID list of Afadin proximal proteins in HEK293 cells

File Name: Supplementary Data 2
Description: BioID list of PAK4, Cdc42 proximal proteins and PAK4 affinity in U2OS cells.

File Name: Supplementary Data 3
Description: Putative PAK4 substrates by phospho-proteomic analysis.
