# Supplementary material for: Proximity proteomics identifies PAK4 as a component of Afadin–Nectin junctions
Source: Nat Commun. 2021 Sep 7;12:5315. doi: 10.1038/s41467-021-25011-w (PMC8423818; doi:10.1038/s41467-021-25011-w)
Supplement: Supplementary file 7 — Reporting Summary [file 41467_2021_25011_MOESM7_ESM.pdf]

nature research

Corresponding author(s): Edward Manser

Last updated by author(s): May 30, 2021

Reporting Summary

Nature Research wishes to improve the reproducibility of the work that we publish. This form provides structure for consistency and transparency in reporting. For further information on Nature Research policies, see our Editorial Policies and the Editorial Policy Checklist.

Statistics

For all statistical analyses, confirm that the following items are present in the figure legend, table legend, main text, or Methods section.

n/a	Confirmed	
☐	☒	The exact sample size (*n*) for each experimental group/condition, given as a discrete number and unit of measurement
☐	☒	A statement on whether measurements were taken from distinct samples or whether the same sample was measured repeatedly
☐	☒	The statistical test(s) used AND whether they are one- or two-sided<br>*Only common tests should be described solely by name; describe more complex techniques in the Methods section.*
☒	☐	A description of all covariates tested
☒	☐	A description of any assumptions or corrections, such as tests of normality and adjustment for multiple comparisons
☐	☒	A full description of the statistical parameters including central tendency (e.g. means) or other basic estimates (e.g. regression coefficient) AND variation (e.g. standard deviation) or associated estimates of uncertainty (e.g. confidence intervals)
☐	☒	For null hypothesis testing, the test statistic (e.g. *F*, *t*, *r*) with confidence intervals, effect sizes, degrees of freedom and *P* value noted<br>*Give P values as exact values whenever suitable.*
☒	☐	For Bayesian analysis, information on the choice of priors and Markov chain Monte Carlo settings
☒	☐	For hierarchical and complex designs, identification of the appropriate level for tests and full reporting of outcomes
☒	☐	Estimates of effect sizes (e.g. Cohen's *d*, Pearson's *r*), indicating how they were calculated

Our web collection on statistics for biologists contains articles on many of the points above.

Software and code

Policy information about availability of computer code

Data collection	Q Exactive™ Hybrid Quadrupole-Orbitrap Mass Spectrometer Controlling Software V 2.0.
Data analysis	MaxQuant (Version 1.3) was used for MS analysis as described in the paper. Cell image analysis was performed with ImageJ.<br>Graph Pad Prism 8.4.1 (Graph Pad Software) was used to graph data and to perform statistical analyses.

For manuscripts utilizing custom algorithms or software that are central to the research but not yet described in published literature, software must be made available to editors and reviewers. We strongly encourage code deposition in a community repository (e.g. GitHub). See the Nature Research guidelines for submitting code & software for further information.

Data

Policy information about availability of data

All manuscripts must include a data availability statement. This statement should provide the following information, where applicable:

- Accession codes, unique identifiers, or web links for publicly available datasets
- A list of figures that have associated raw data
- A description of any restrictions on data availability

Figure 1C Complete SILAC MS data can be found in Supplementary Table 2
Figure 2B Complete SILAC BioID data is listed in Supplementary Table 1
Figure 4B/C/D Scatter plots based on micrograph data (to be provided)
Figure 6B SILAC MS data is listed in Supplementary Table T3
Figure 7B Radiolabelled peptide spot data (autoradiograph to be provided).
Supplementary Figure S2. Scatter plots based on micrograph data (to be provided)

Supplementary Figure S4. Scatter plots based on micrograph data (to be provided)
Afadin BioID data derived from https://cell-map.org/

Field-specific reporting

Please select the one below that is the best fit for your research. If you are not sure, read the appropriate sections before making your selection.

☒ Life sciences ☐ Behavioural & social sciences ☐ Ecological, evolutionary & environmental sciences

For a reference copy of the document with all sections, see nature.com/documents/nr-reporting-summary-flat.pdf

Life sciences study design

All studies must disclose on these points even when the disclosure is negative.

Sample size	For MS data the SILAC enrichment ratio (shown in the figures and tables) is based on a minimum of 3 measurements. The errors that are given in the Tables indicate that the results are statistically significant.
Data exclusions	In MS data non-specific protein contaminants were excluded based on SILAC values < 3 (see figure legends). This criterion was based on our previous work using SILAC BioID to analyse multiple datasets derived from paxillin and Kindlin-2.
Replication	Phospho-proteomic data required measurement of at least 3 phospho-peptides in replicate experiments. Immunofluorescent imaging of cells was carried out in triplicate and pictures shown are representative of results obtained. The use of SiRNA to knockdown target proteins was carried out using at least two duplexes, and the data presented (cf. Fig 5) that the knockdown was not uniform across a given field. Therefore, data replication were successful .
Randomization	No randomization was required since the MS studies use both external (experimental) and internal (isotope labelled) controls. The fields that were imaged and then taken for image analysis were selected randomly,
Blinding	No protocols required blinding. Thresholds for detecting the position of cell junctions were the enrichment of well established junctional markers (using antibody staining), as indicated on each panel and were indistinguishable across different experiments (using the same antibodies).

Reporting for specific materials, systems and methods

We require information from authors about some types of materials, experimental systems and methods used in many studies. Here, indicate whether each material, system or method listed is relevant to your study. If you are not sure if a list item applies to your research, read the appropriate section before selecting a response.

Materials & experimental systems

n/a	Involved in the study	
☐	☒	Antibodies
☐	☒	Eukaryotic cell lines
☒	☐	Palaeontology and archaeology
☒	☐	Animals and other organisms
☒	☐	Human research participants
☒	☐	Clinical data
☒	☐	Dual use research of concern

Methods

n/a	Involved in the study	
☒	☐	ChIP-seq
☒	☐	Flow cytometry
☒	☐	MRI-based neuroimaging

Antibodies

Antibodies used	Rabbit anti-PAK4 (Proteintech 14685-1-AP); mouse anti-p120-catenin (Santa Cruz sc-23873); rabbit anti-β-catenin (Cell Signaling #9582S); mouse anti-β-catenin (Santa Cruz sc-7963); anti-LZTS2 (Proteintech 15677-1-AP); anti-Afadin (Sigma A0224); mouse anti-Afadin (R&D Systems MAB78291); rat anti-ZO-1, (Santa Cruz sc-33725); rabbit anti-DLG5 (Abcam ab86783); rabbit anti-Scribble (Santa Cruz sc-28737); rabbit anti-p120-catenin pS320 (Cell Signaling #8016S), mouse β-tubulin (Sigma T4026).
Validation	The anti-PAK4 and anti-Afadin Abs were validated by SiRNA experiments that are presented in this paper. The anti-β-catenin and anti-ZO-1 are widely used junctional markers and were used to assay the location and nature of the junctions in U2OS and MDCK cell lines.

Eukaryotic cell lines

Policy information about cell lines

Cell line source(s)	U2OS (ATCC); MDCK (ATCC).

Authentication	MDCK was validated by proteomics as dog (ie dog-unique sequences detected).
Mycoplasma contamination	Both lines are tested for mycoplasma contamination and found to be negative.
Commonly misidentified lines (See ICLAC register)	No commonly misidentified cell lines were used.
