# Supplementary figures and images for: Proximity proteomics identifies PAK4 as a component of Afadin–Nectin junctions
Source: Nat Commun. 2021 Sep 7;12:5315. doi: 10.1038/s41467-021-25011-w (PMC8423818; doi:10.1038/s41467-021-25011-w)

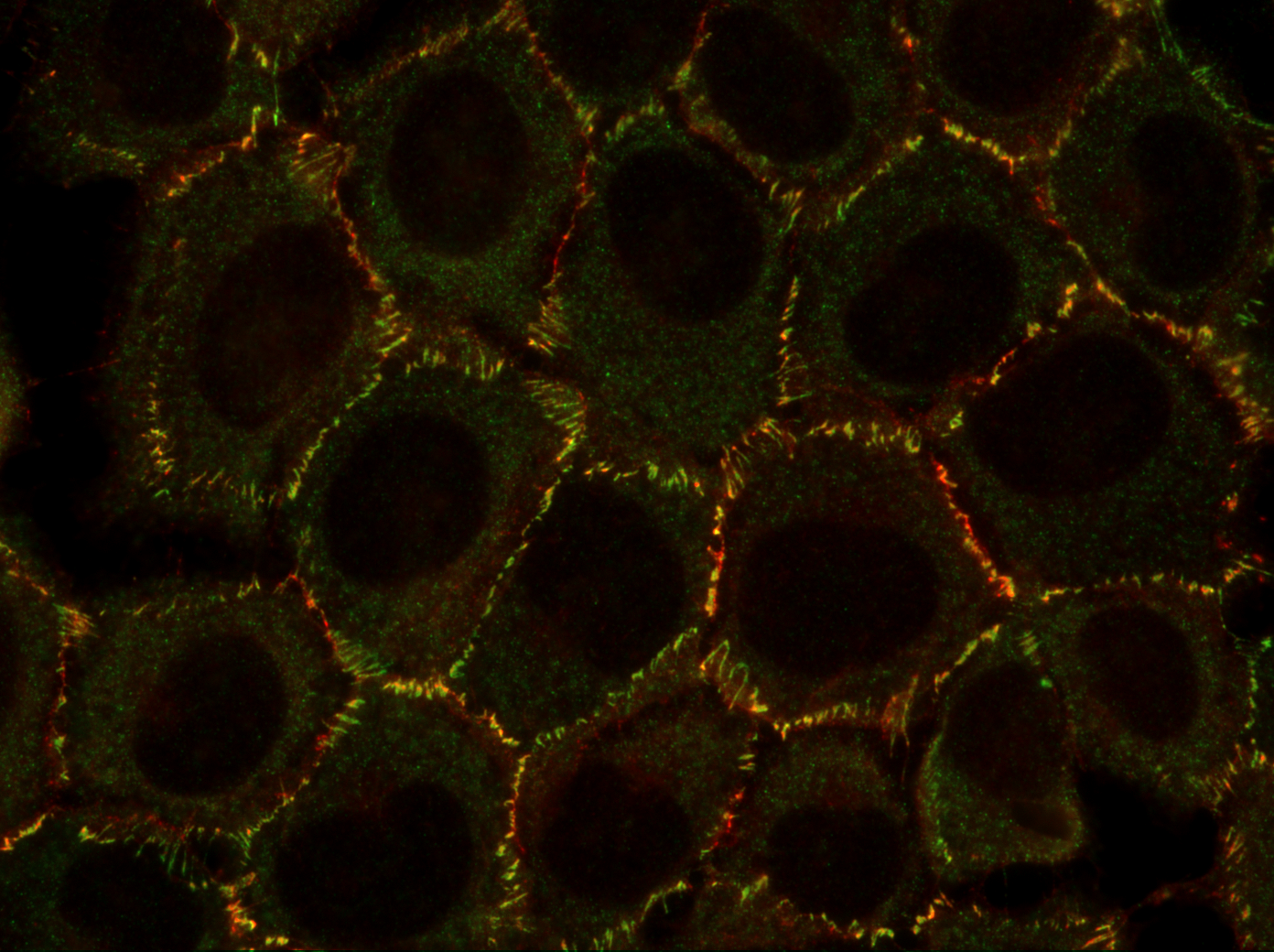

Supplement: Supplementary file 8 — Source Data [file 41467_2021_25011_MOESM8_ESM.zip › Source Data Images/Figure S2/Afadin p120 C2.tif]

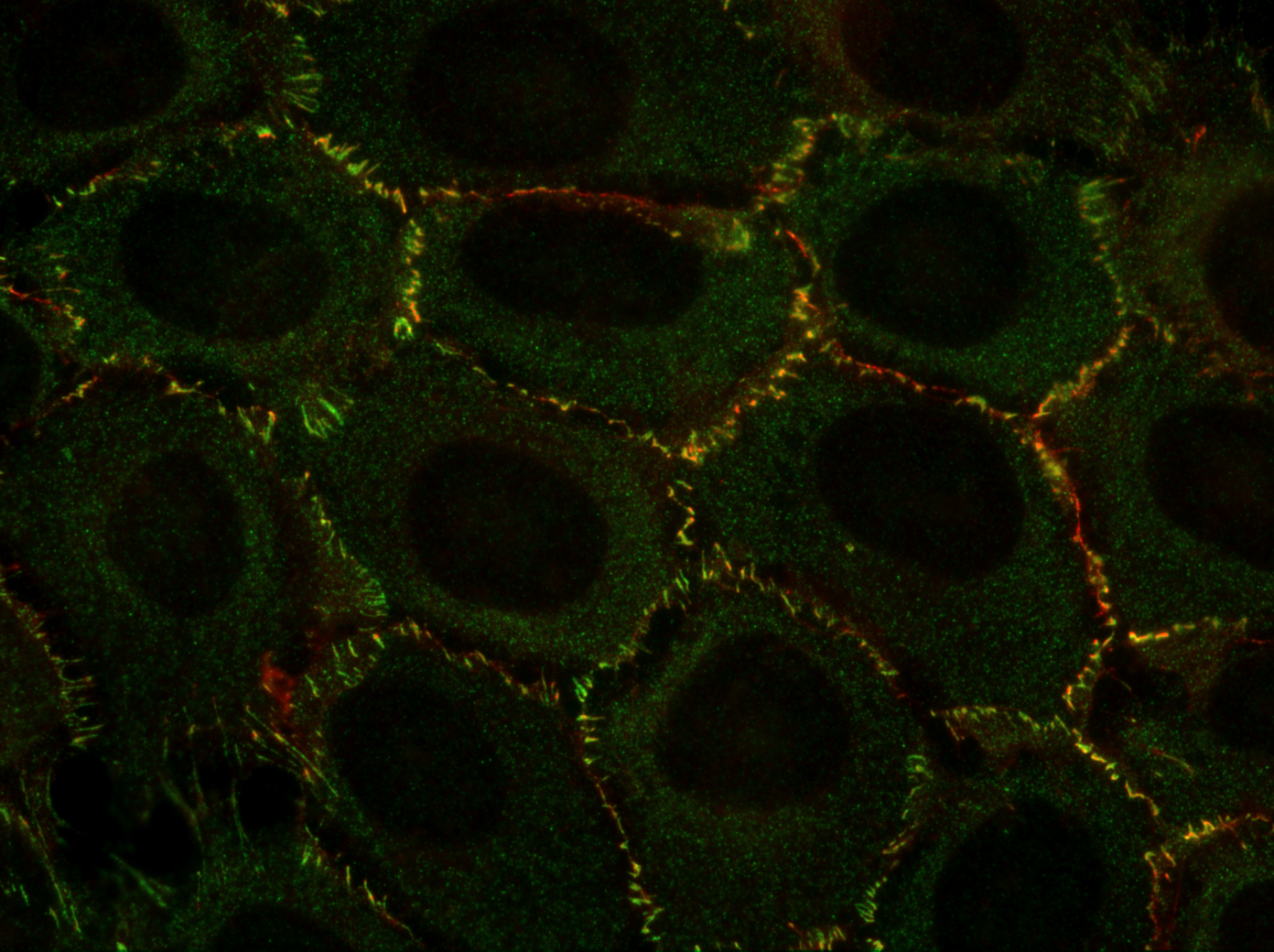

Supplement: Supplementary file 8 — Source Data [file 41467_2021_25011_MOESM8_ESM.zip › Source Data Images/Figure S2/Afadin p120 C3.tif]

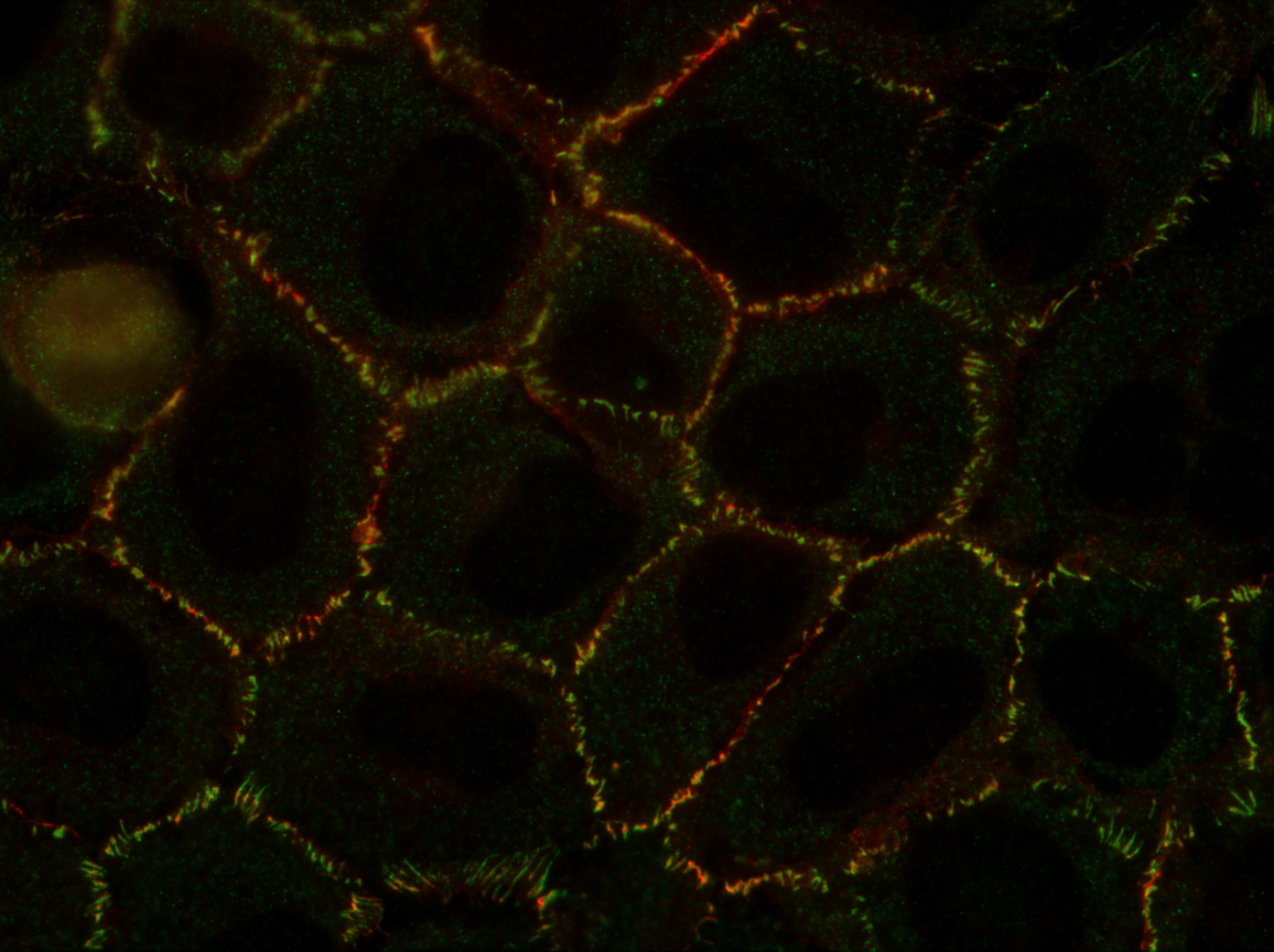

Supplement: Supplementary file 8 — Source Data [file 41467_2021_25011_MOESM8_ESM.zip › Source Data Images/Figure S2/Afadin p120 C5.tif]

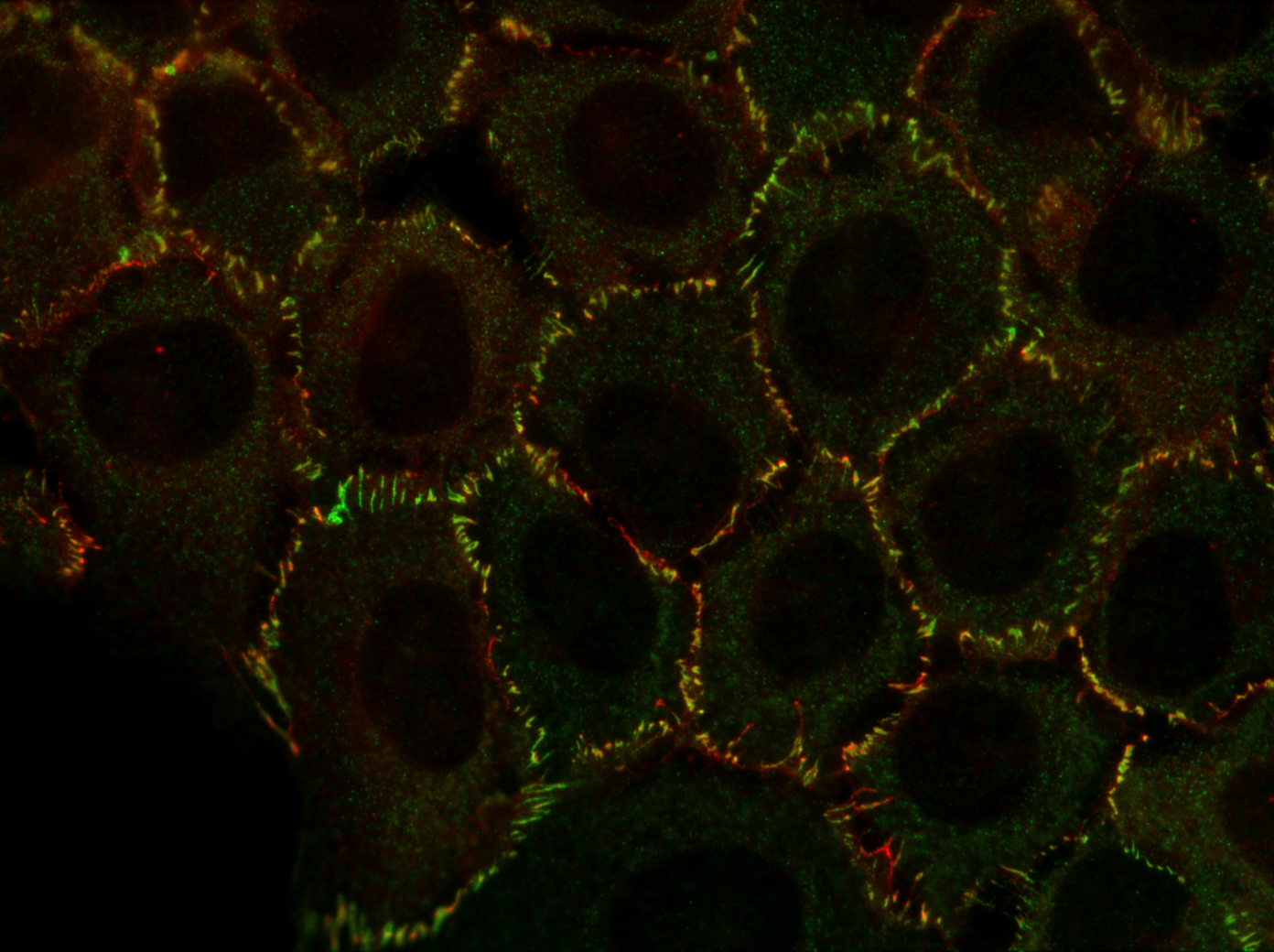

Supplement: Supplementary file 8 — Source Data [file 41467_2021_25011_MOESM8_ESM.zip › Source Data Images/Figure S2/Afadin p120 C6.tif]

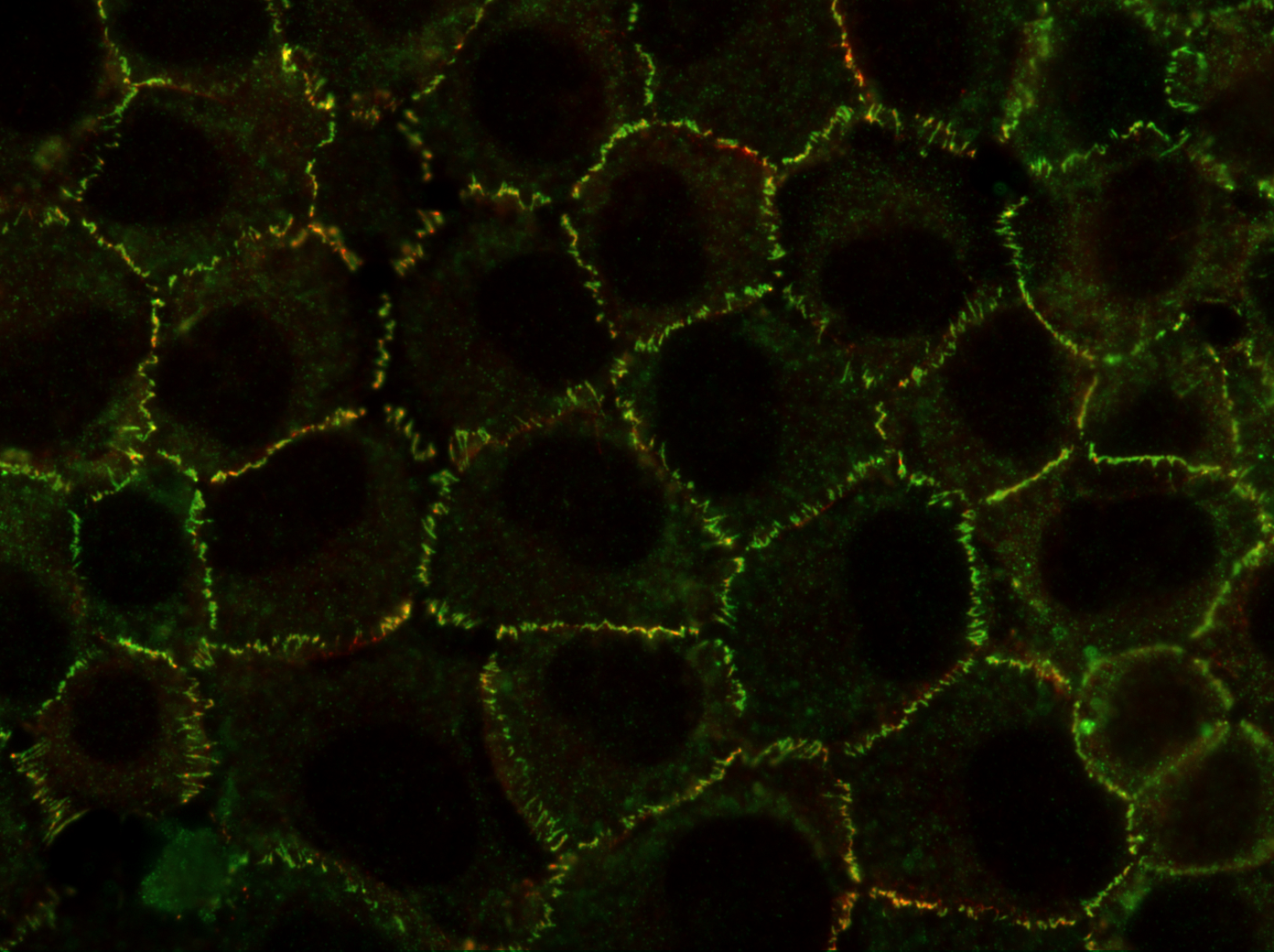

Supplement: Supplementary file 8 — Source Data [file 41467_2021_25011_MOESM8_ESM.zip › Source Data Images/Figure S2/Afadin p120 PAKi1.tif]

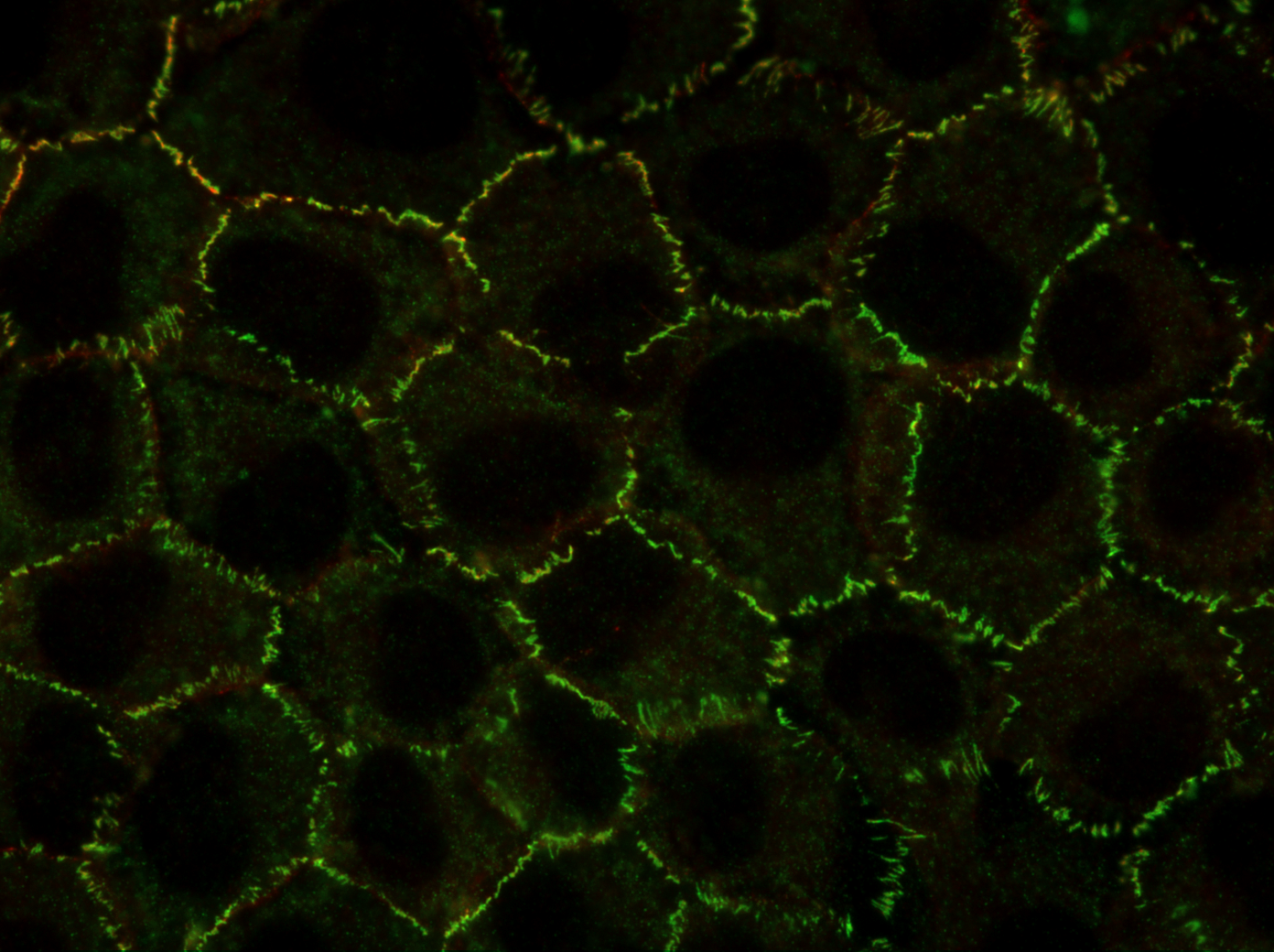

Supplement: Supplementary file 8 — Source Data [file 41467_2021_25011_MOESM8_ESM.zip › Source Data Images/Figure S2/Afadin p120 PAKi2.tif]

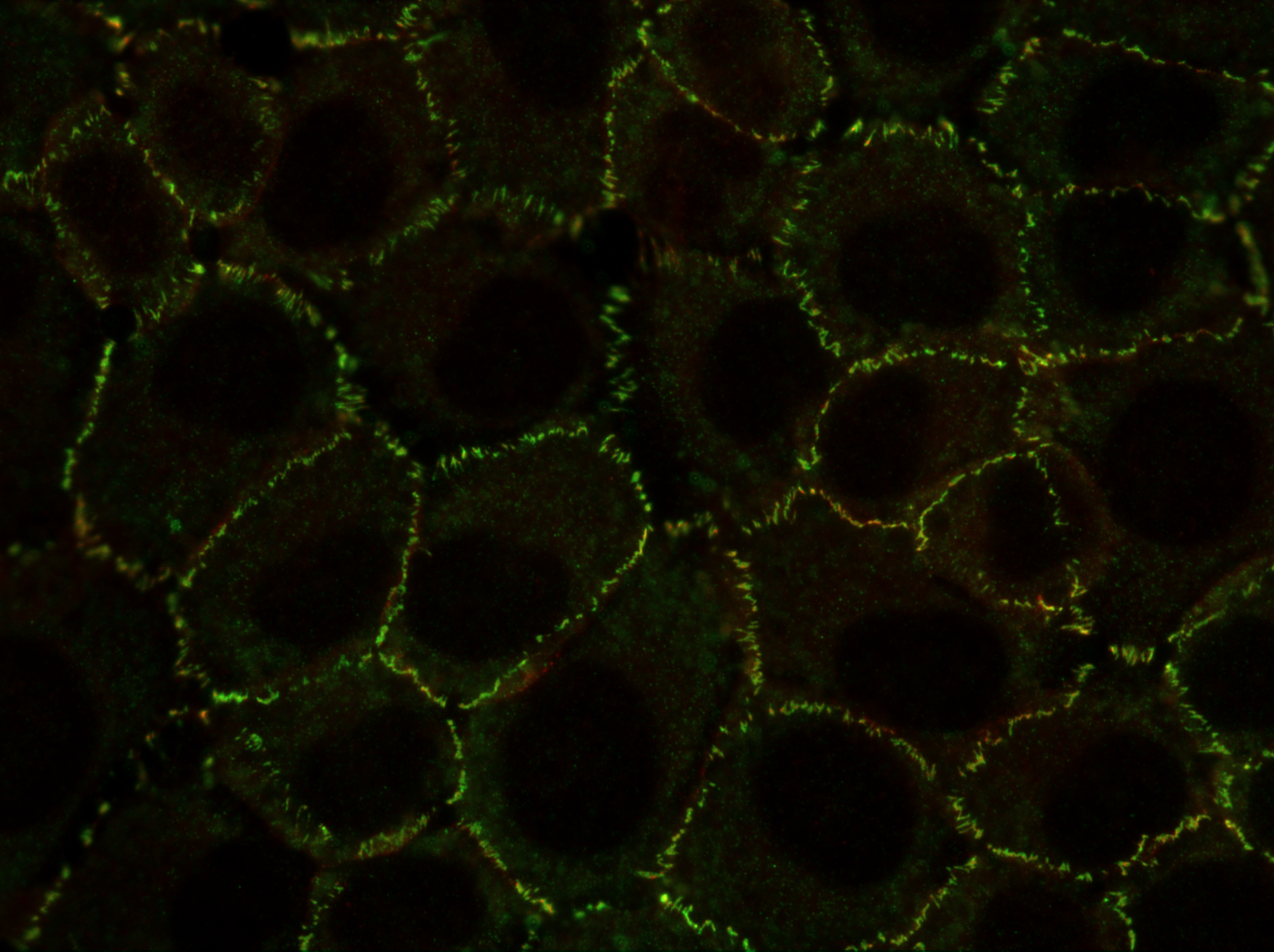

Supplement: Supplementary file 8 — Source Data [file 41467_2021_25011_MOESM8_ESM.zip › Source Data Images/Figure S2/Afadin p120 PAKi5.tif]

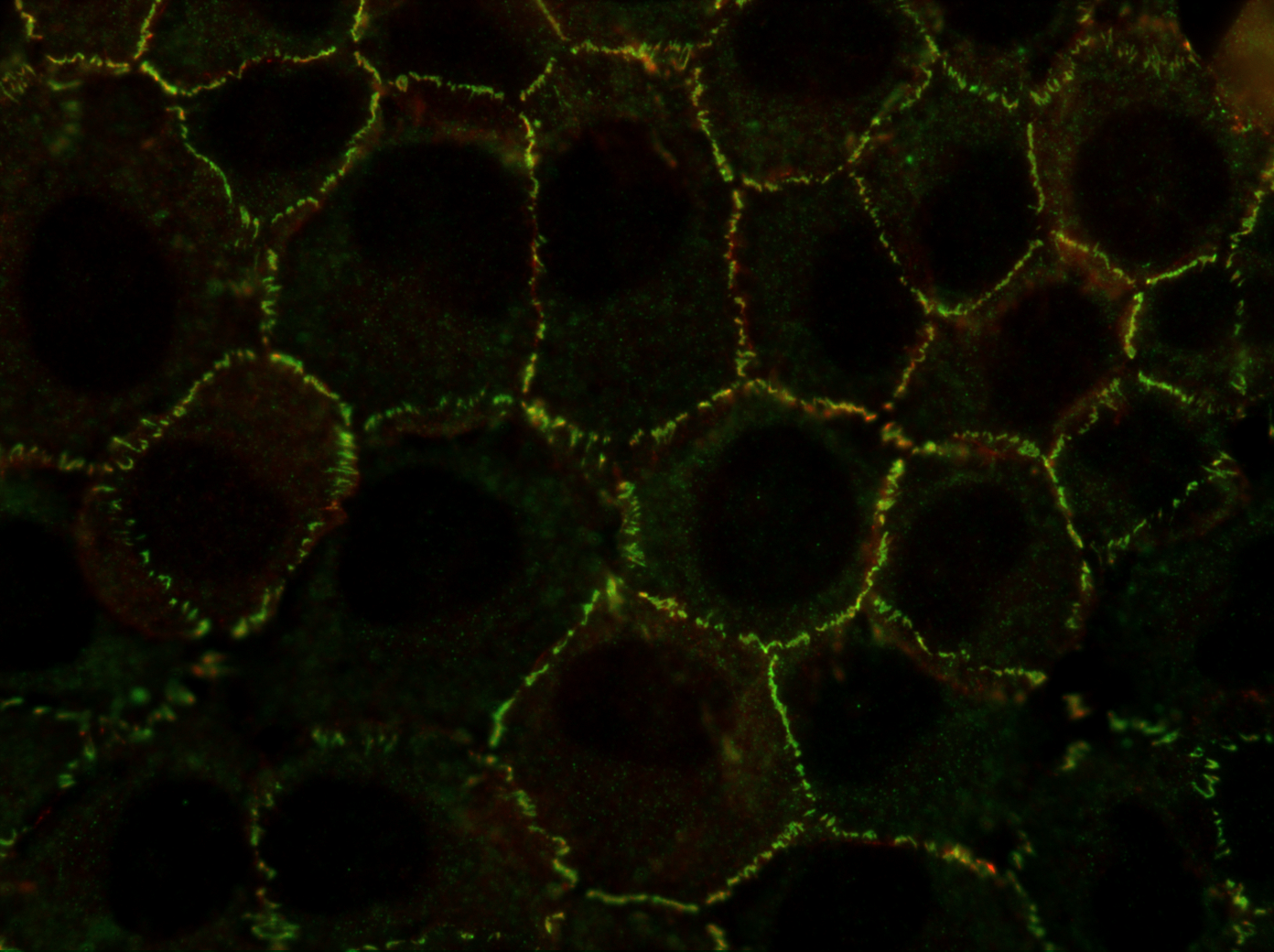

Supplement: Supplementary file 8 — Source Data [file 41467_2021_25011_MOESM8_ESM.zip › Source Data Images/Figure S2/Afadin p120 PAKi6.tif]

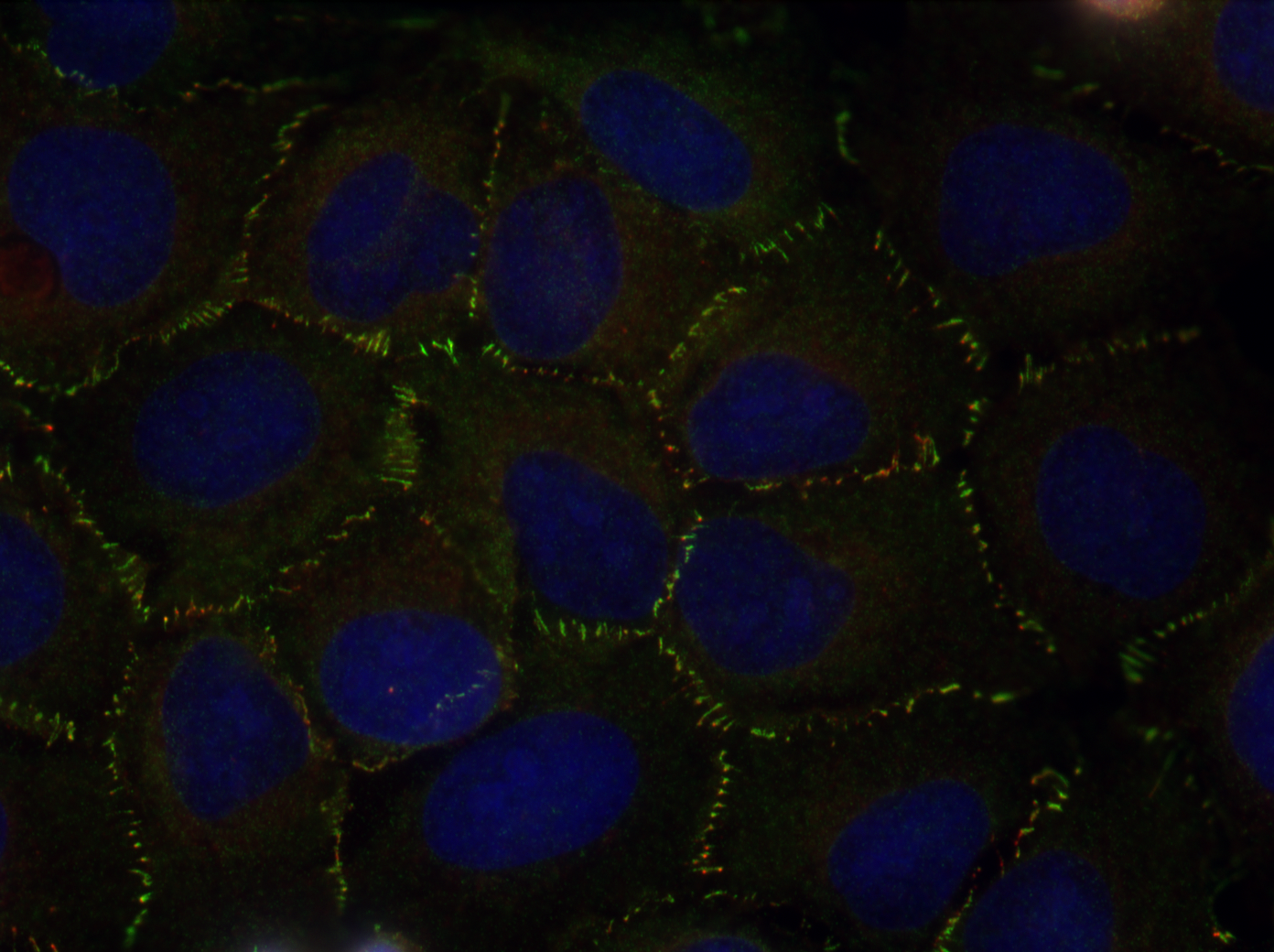

Supplement: Supplementary file 8 — Source Data [file 41467_2021_25011_MOESM8_ESM.zip › Source Data Images/Figure S2/PAK4 p120 C2.tif]

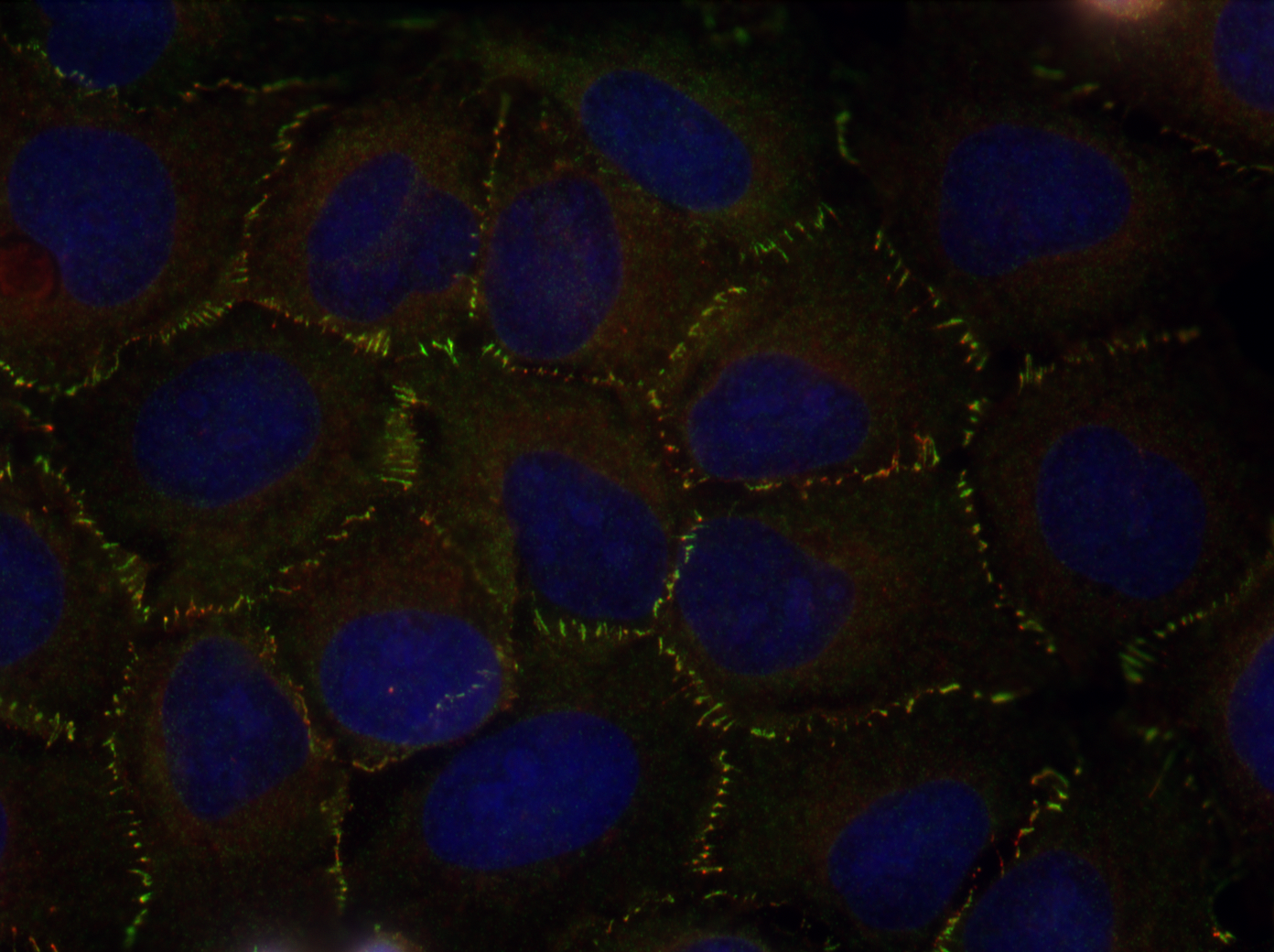

Supplement: Supplementary file 8 — Source Data [file 41467_2021_25011_MOESM8_ESM.zip › Source Data Images/Figure S2/PAK4 p120 C2.tiff]

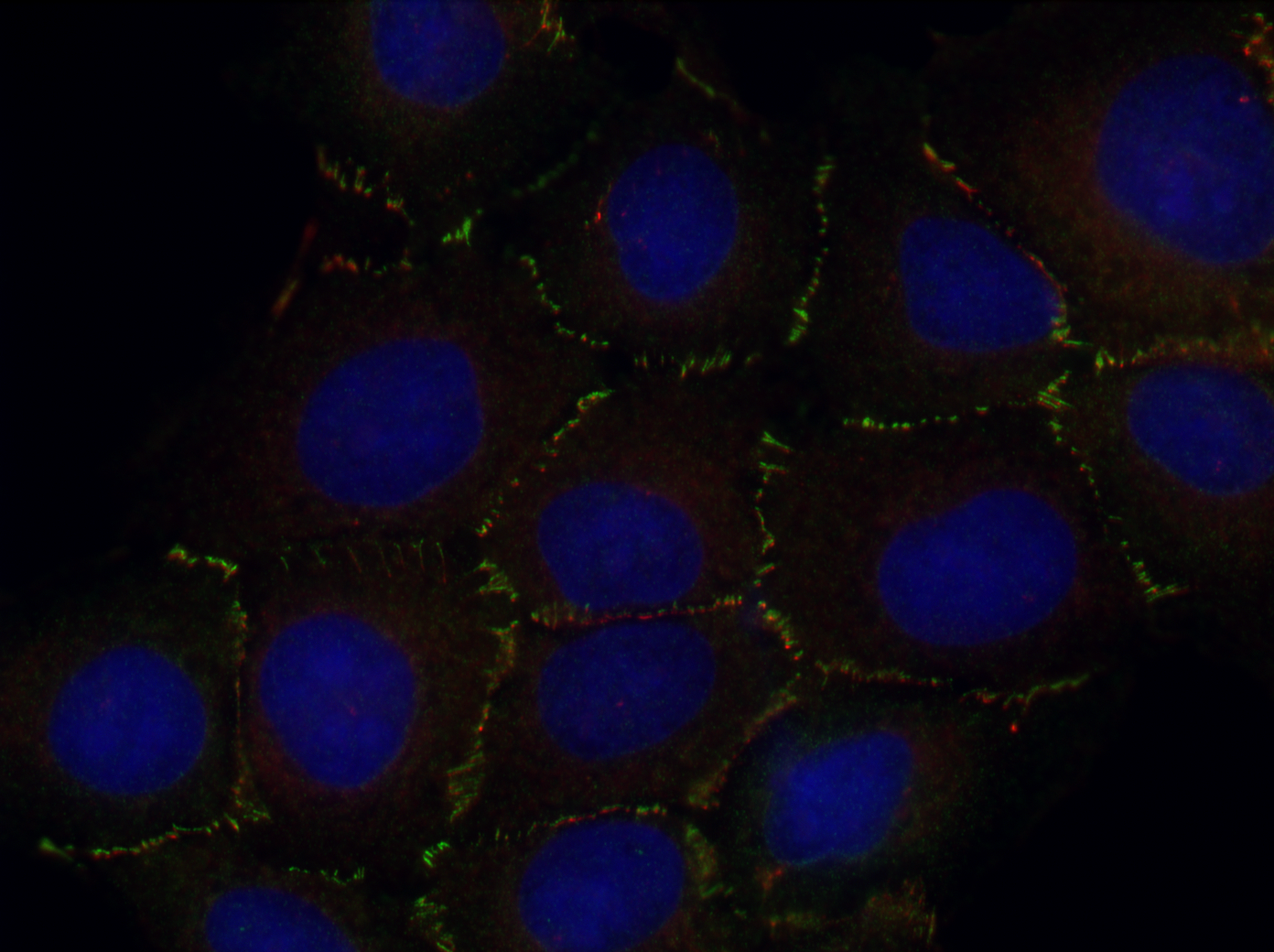

Supplement: Supplementary file 8 — Source Data [file 41467_2021_25011_MOESM8_ESM.zip › Source Data Images/Figure S2/PAK4 p120 C3.tif]

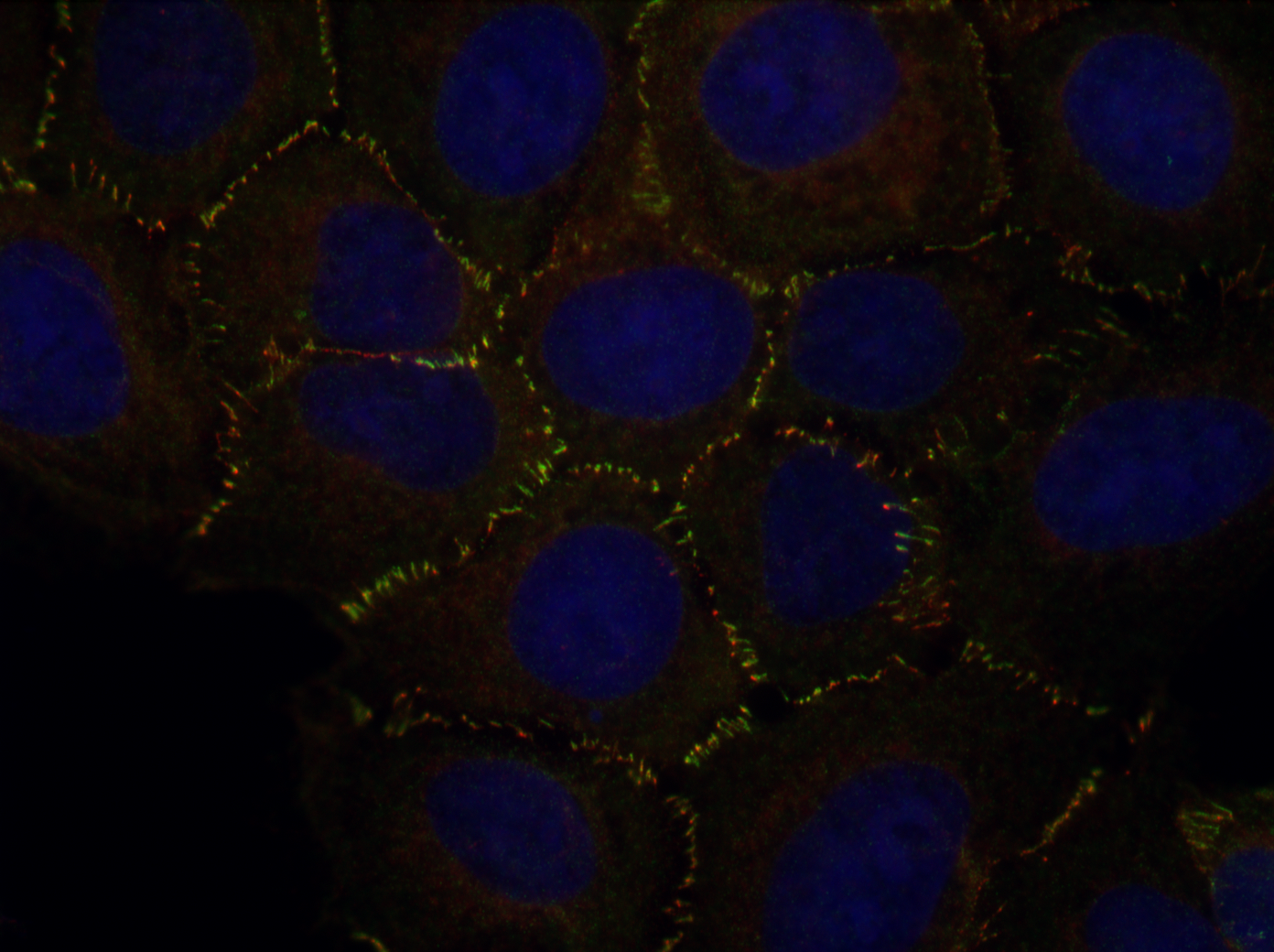

Supplement: Supplementary file 8 — Source Data [file 41467_2021_25011_MOESM8_ESM.zip › Source Data Images/Figure S2/PAK4 p120 C5.tiff]

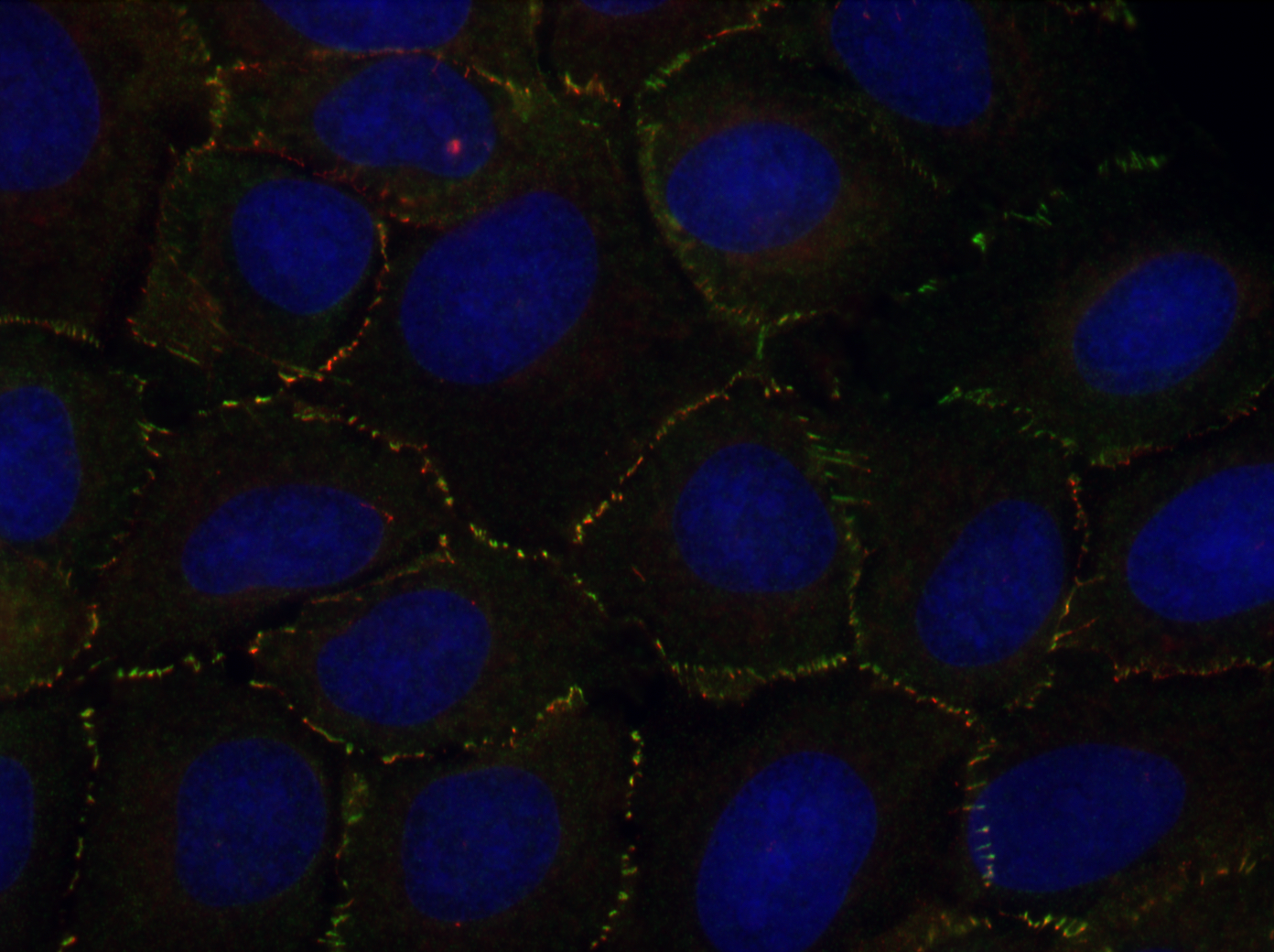

Supplement: Supplementary file 8 — Source Data [file 41467_2021_25011_MOESM8_ESM.zip › Source Data Images/Figure S2/PAK4 p120 C6.tif]

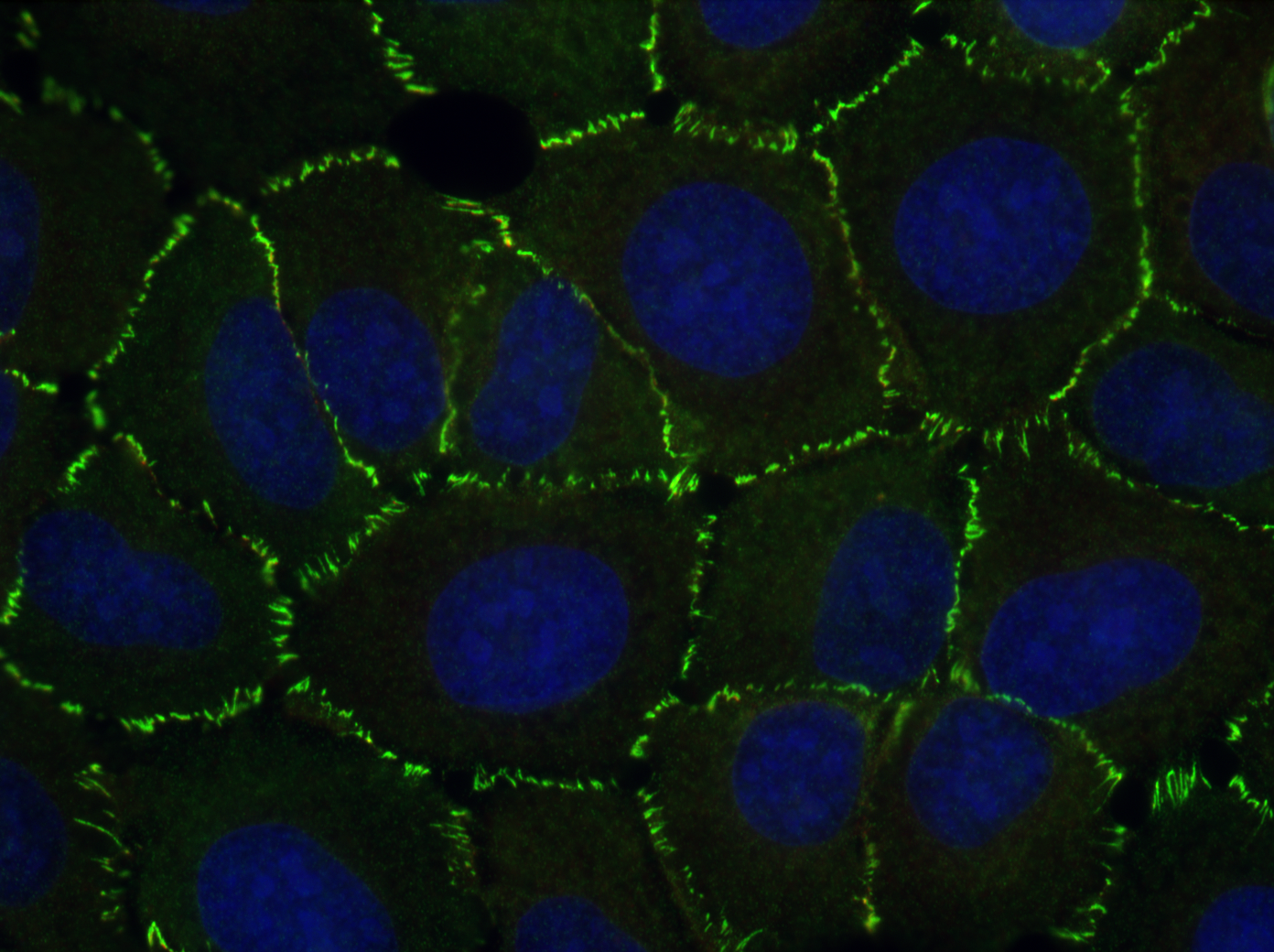

Supplement: Supplementary file 8 — Source Data [file 41467_2021_25011_MOESM8_ESM.zip › Source Data Images/Figure S2/PAK4 p120 PF2.tif]

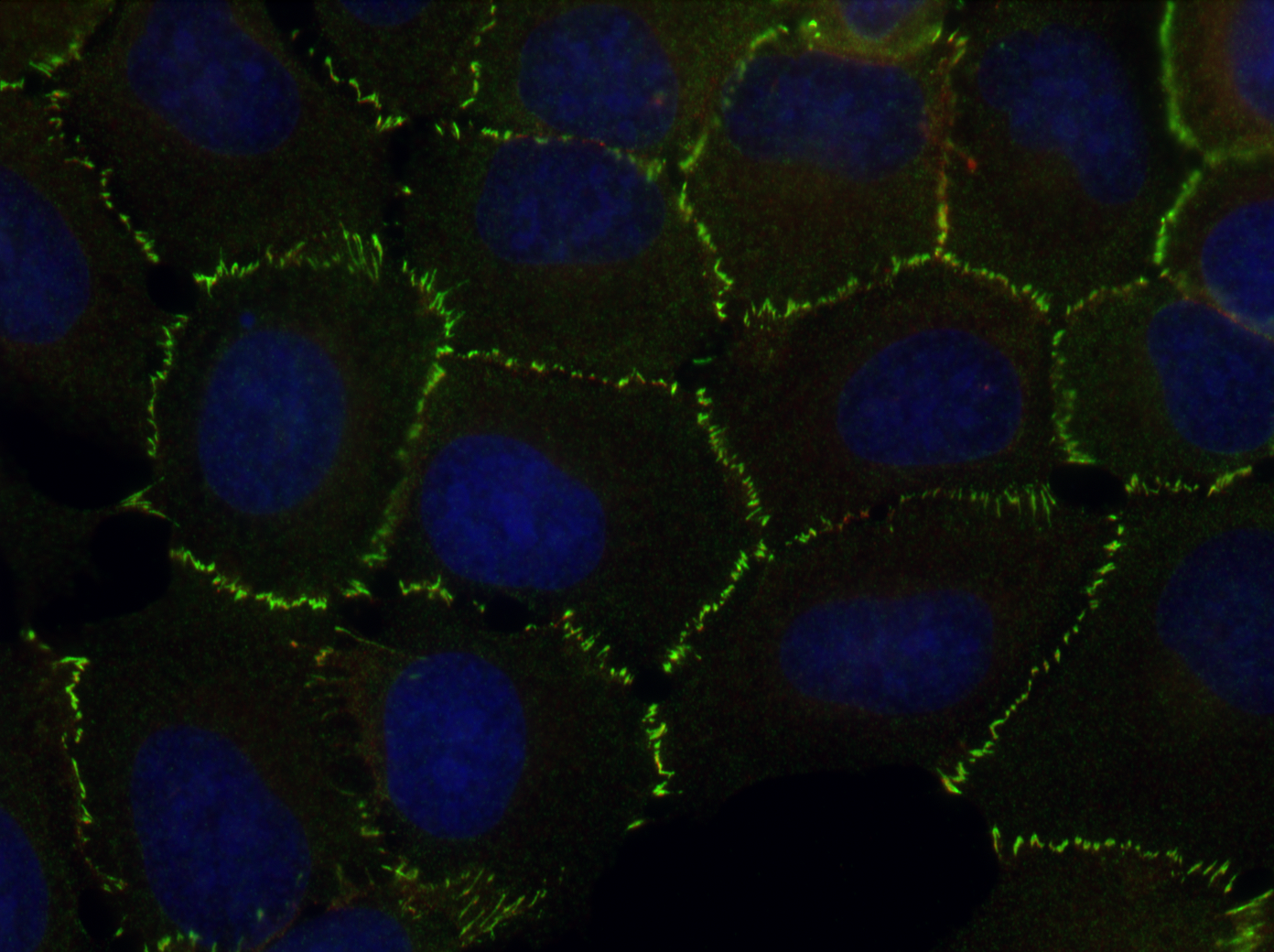

Supplement: Supplementary file 8 — Source Data [file 41467_2021_25011_MOESM8_ESM.zip › Source Data Images/Figure S2/PAK4 p120 PF3.tiff]

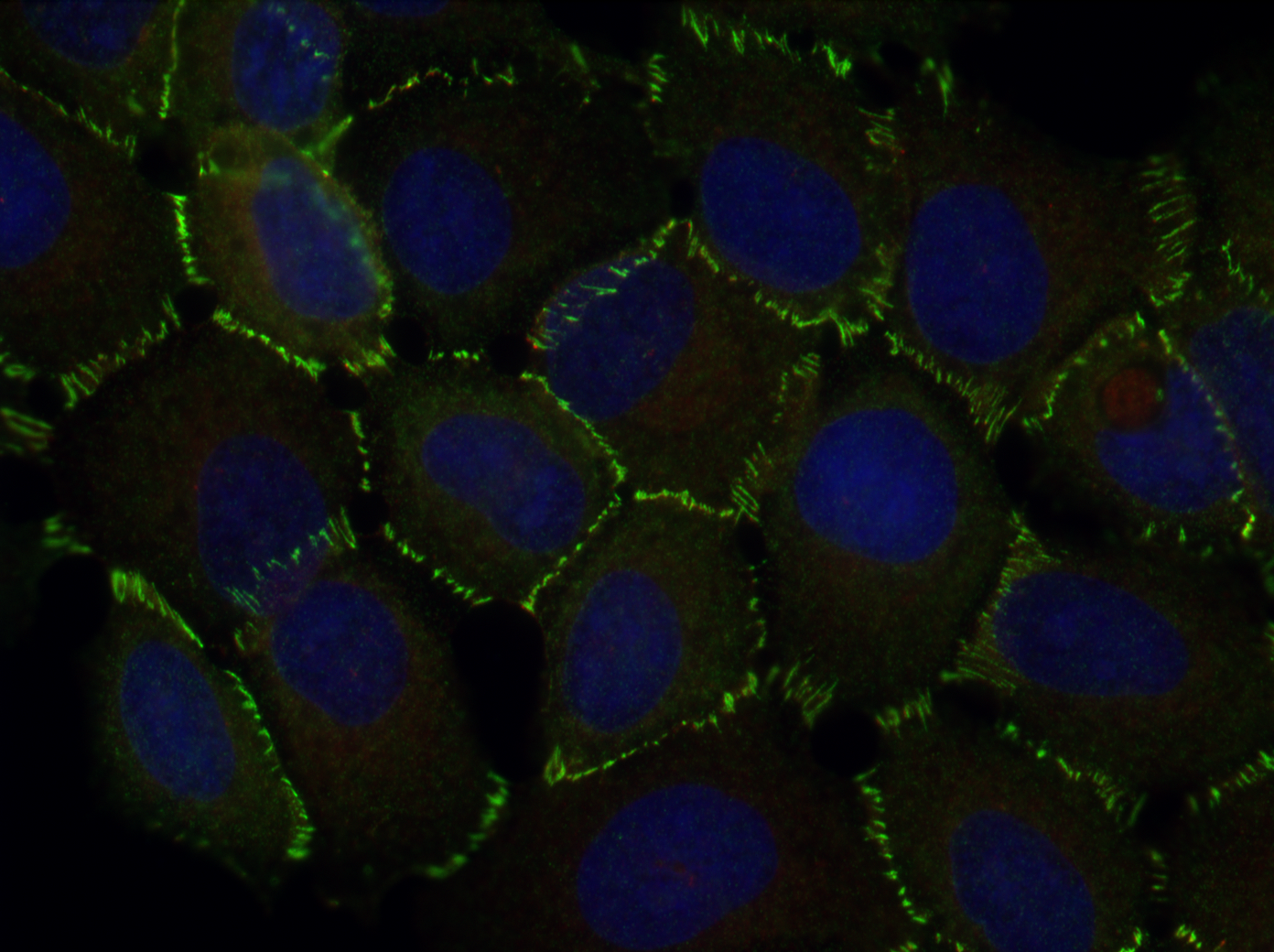

Supplement: Supplementary file 8 — Source Data [file 41467_2021_25011_MOESM8_ESM.zip › Source Data Images/Figure S2/PAK4 p120 PF4.tiff]

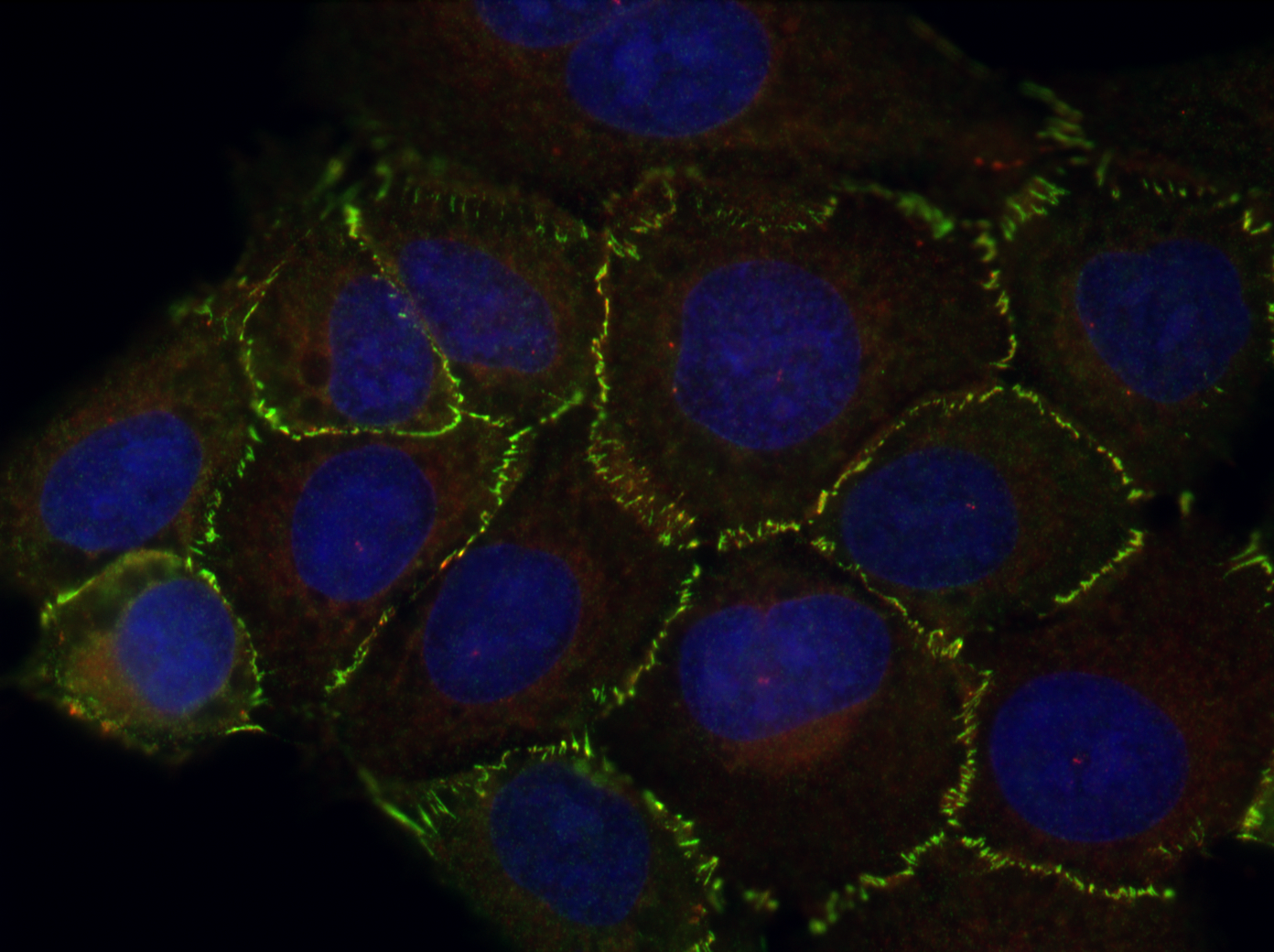

Supplement: Supplementary file 8 — Source Data [file 41467_2021_25011_MOESM8_ESM.zip › Source Data Images/Figure S2/PAK4 p120 PF5.tif]

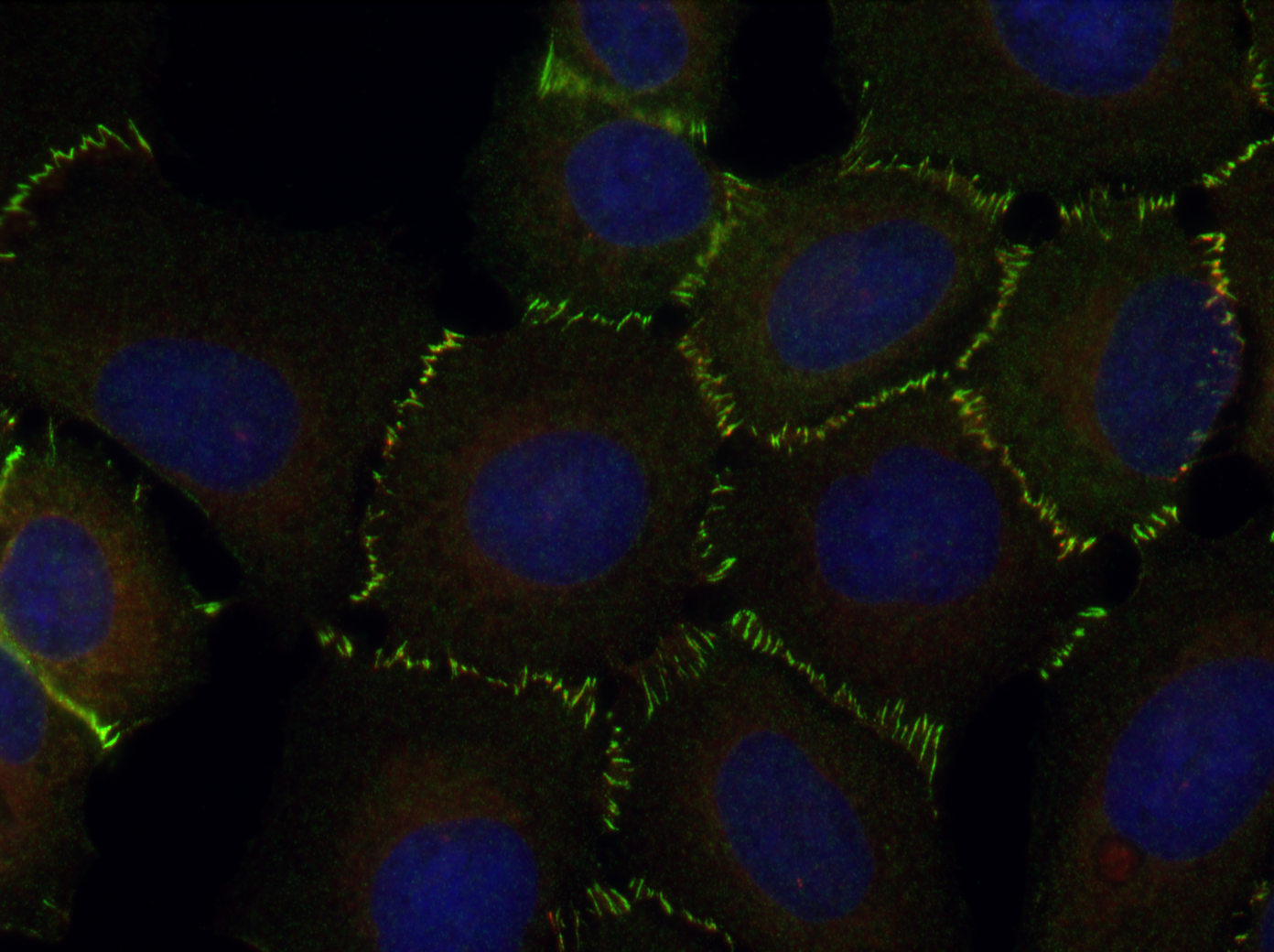

Supplement: Supplementary file 8 — Source Data [file 41467_2021_25011_MOESM8_ESM.zip › Source Data Images/Figure S2/PAK4 p120 PF6.tif]

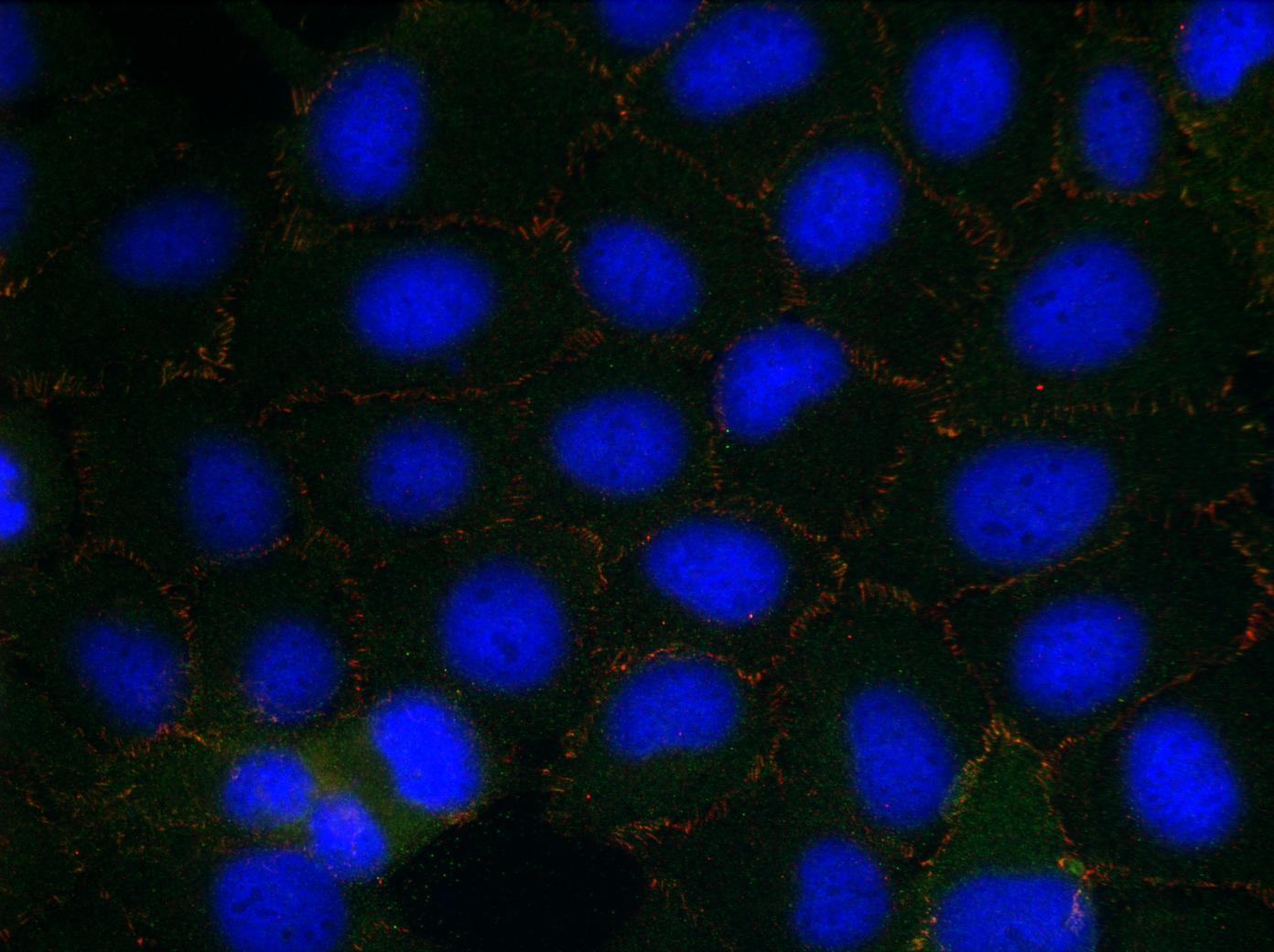

Supplement: Supplementary file 8 — Source Data [file 41467_2021_25011_MOESM8_ESM.zip › Source Data Images/Figure S4b/U2OS x40 siP4 rP4 mAF6 A.tif]

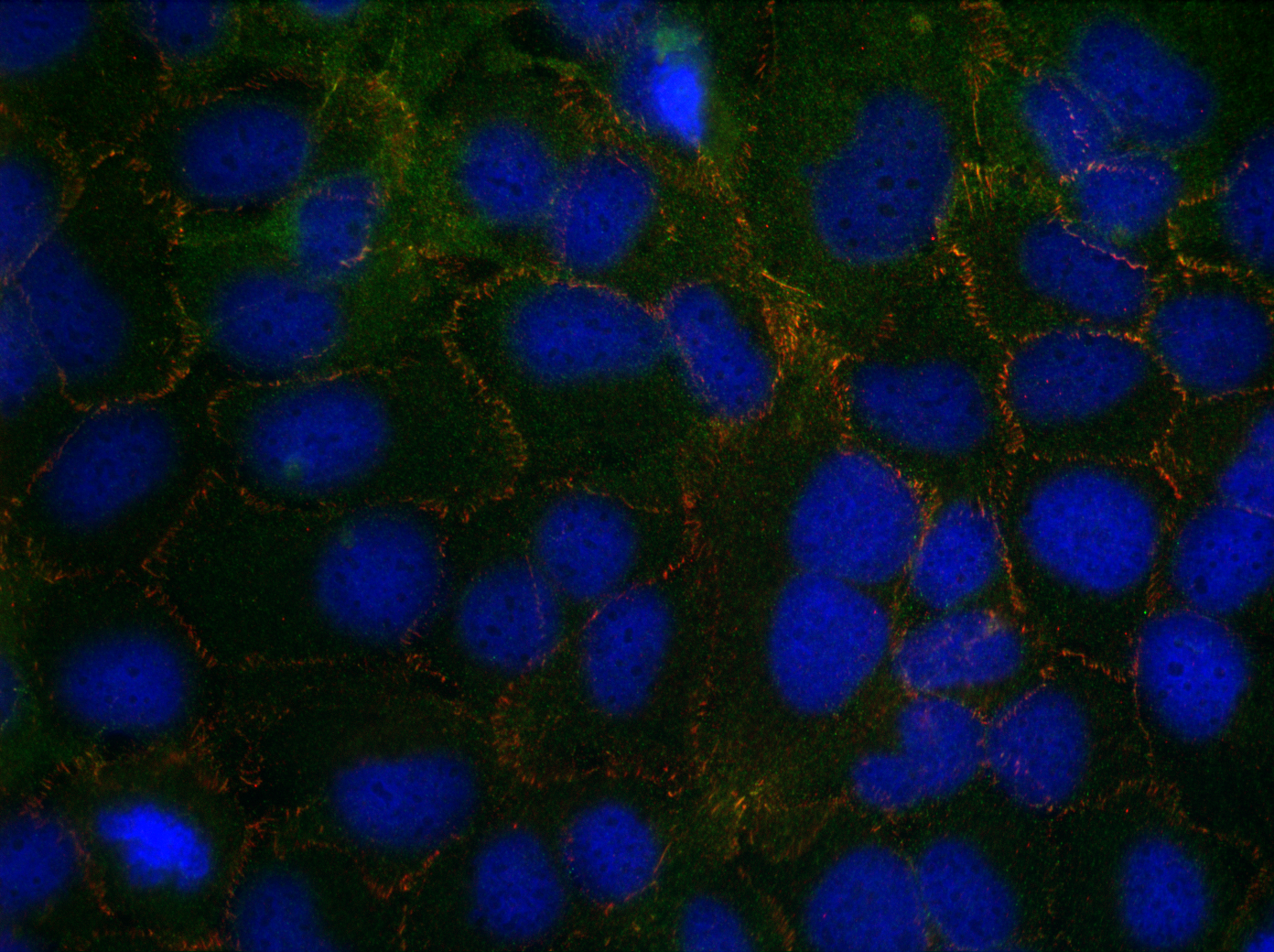

Supplement: Supplementary file 8 — Source Data [file 41467_2021_25011_MOESM8_ESM.zip › Source Data Images/Figure S4b/U2OS x40 siP4 rP4 mAF6 B.tif]

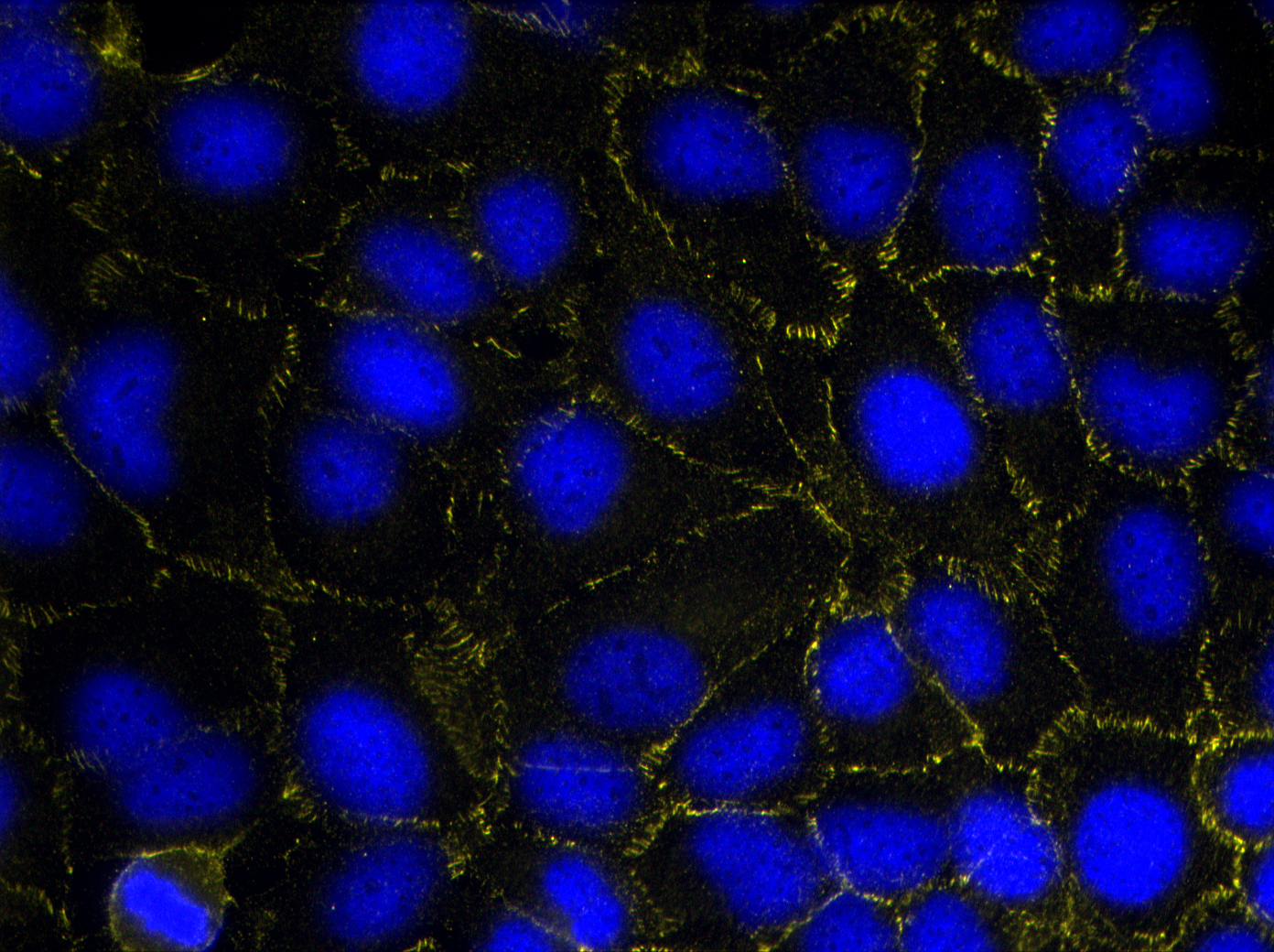

Supplement: Supplementary file 8 — Source Data [file 41467_2021_25011_MOESM8_ESM.zip › Source Data Images/Figure S4b/U2OS x40 siP4 rP4 mAF6 C.tif]

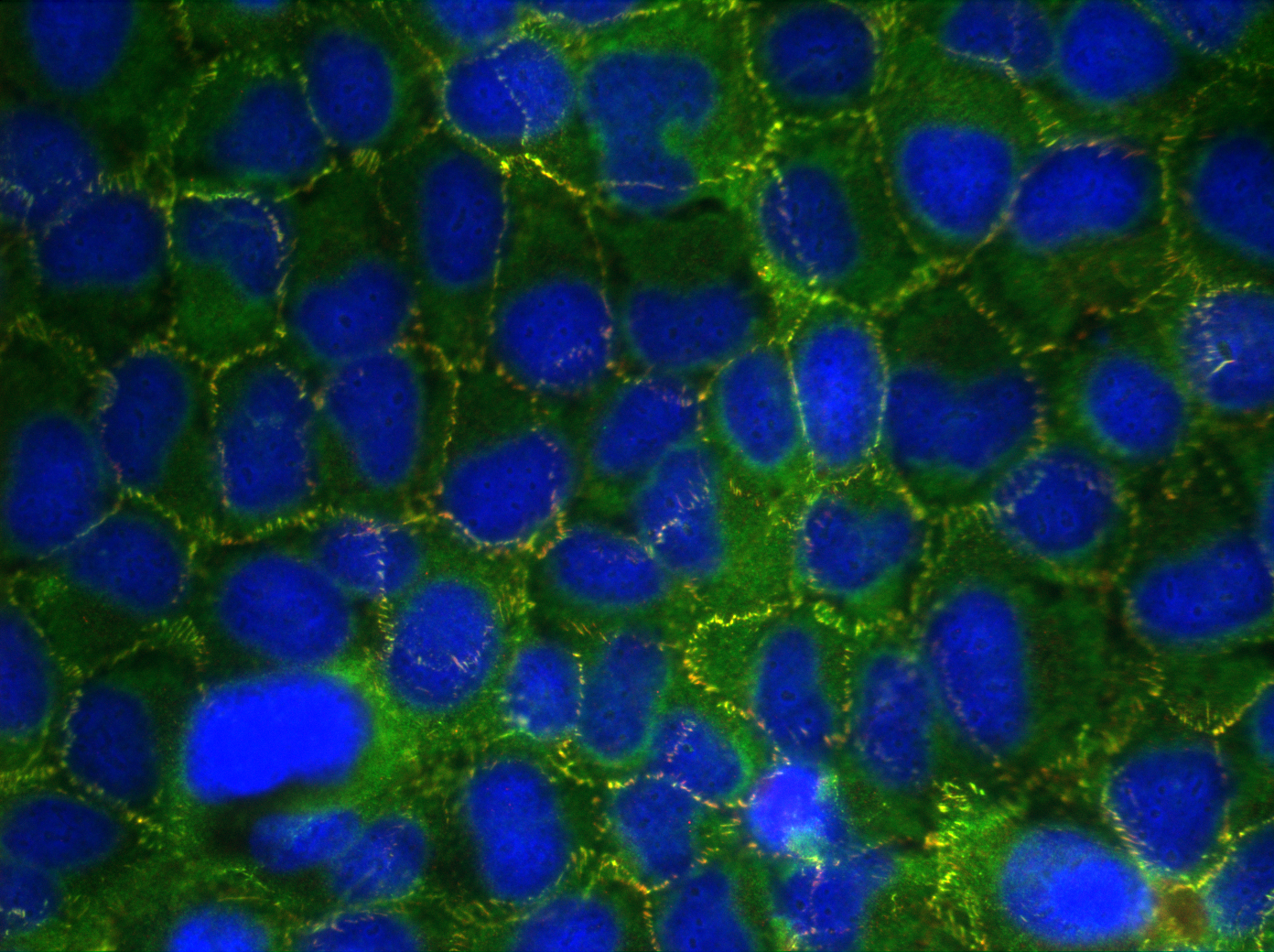

Supplement: Supplementary file 8 — Source Data [file 41467_2021_25011_MOESM8_ESM.zip › Source Data Images/Figure S4b/U2OS x40 siSCR rP4 mAF6 A.tif]

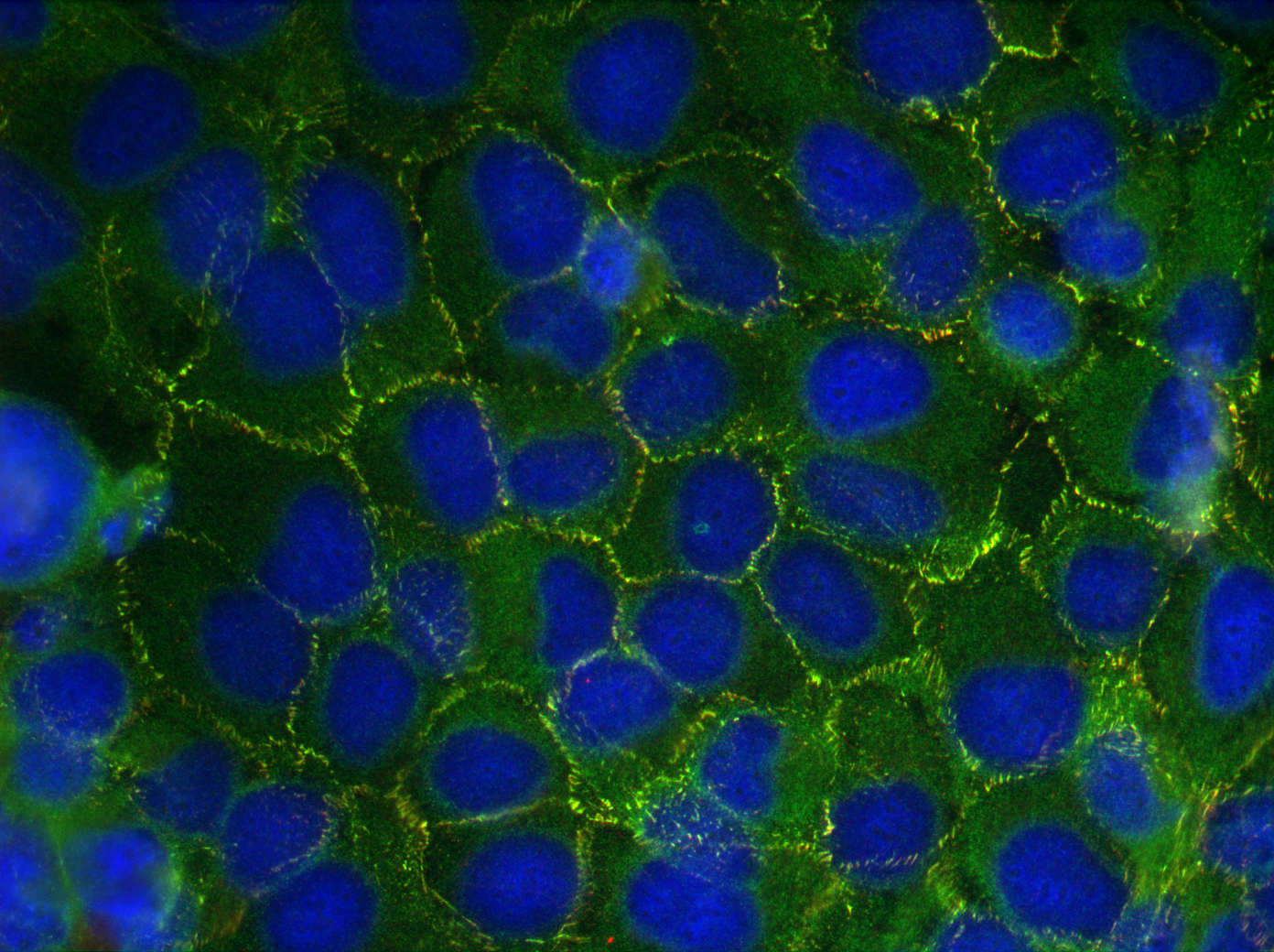

Supplement: Supplementary file 8 — Source Data [file 41467_2021_25011_MOESM8_ESM.zip › Source Data Images/Figure S4b/U2OS x40 siSCR rP4 mAF6 B.tif]

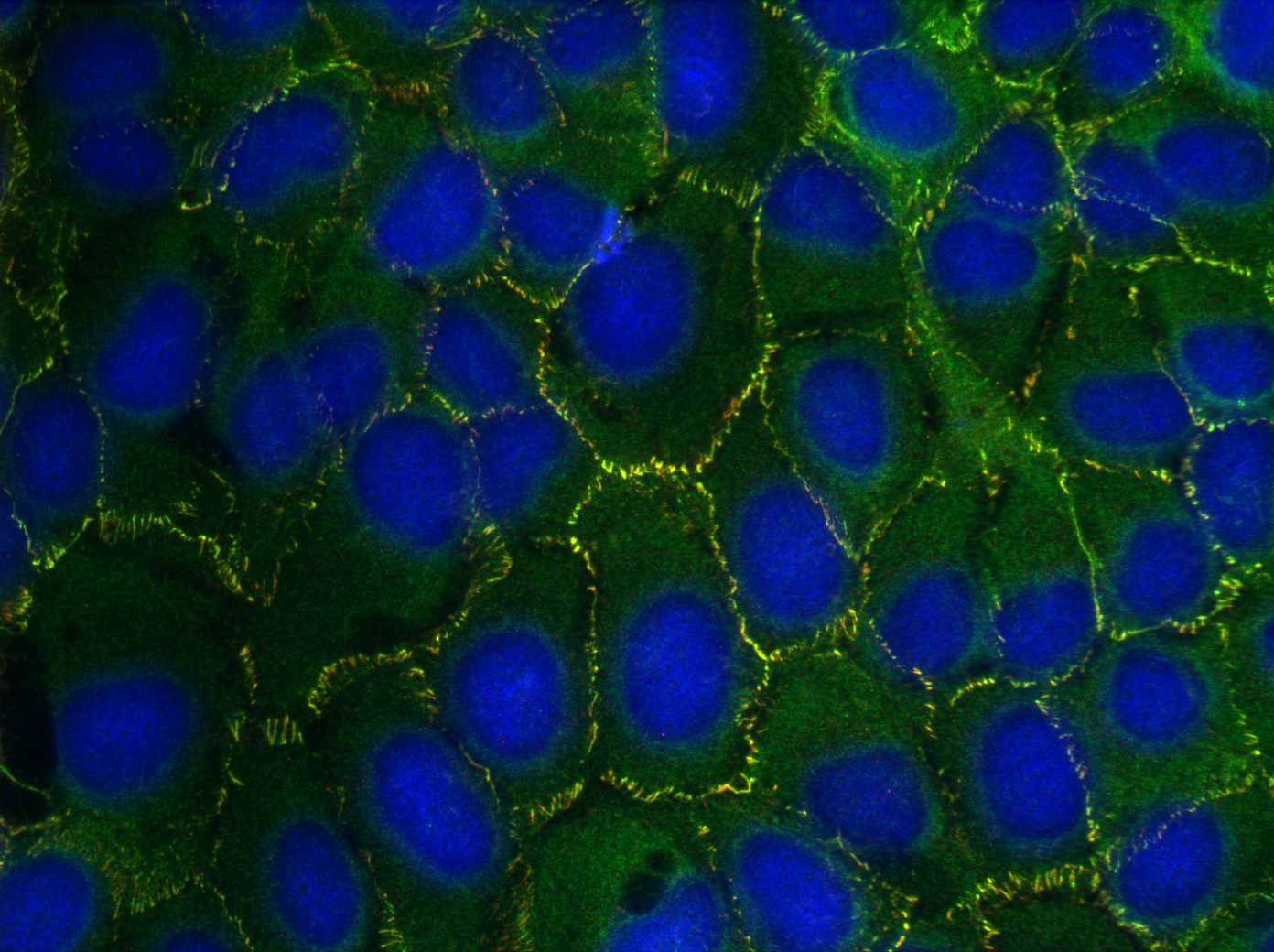

Supplement: Supplementary file 8 — Source Data [file 41467_2021_25011_MOESM8_ESM.zip › Source Data Images/Figure S4b/U2OS x40 siSCR rP4 mAF6 C.tif]
